# Cation Hydrophobicity Effects on Protein Solvation in Aqueous Ionic Liquids

**DOI:** 10.1021/acs.jpcb.5c00779

**Published:** 2025-05-29

**Authors:** Vinicius Piccoli, Leandro Martínez

**Affiliations:** Institute of Chemistry and Center for Computing in Engineering & Science, 28132 Universidade Estadual de Campinas (UNICAMP), Campinas, SP 13083-872, Brazil

## Abstract

This study examines the influence of cation hydrophobicity on protein solvation in aqueous solutions of Ionic Liquids. Ubiquitin solvation structures and thermodynamics in systems with 1-ethyl-3-methylimidazolium ([EMIM]^+^) and 1-butyl-3-methylimidazolium ([BMIM]^+^) are studied using molecular dynamics simulations, minimum-distance distribution functions, and the Kirkwood–Buff theory of solvation. At low concentrations, the larger alkyl chain leads to enhanced water exclusion and increased accumulation of [BMIM]^+^ at the protein surface relative to [EMIM]^+^. The preferential solvation, nevertheless, depends on the ionic liquid concentration differently for each cation. As concentrations increase, [BMIM]^+^ relative accumulation decreases relative to [EMIM]^+^. This causes a reversal of cation–protein affinities relative to water, and [EMIM]^+^ displays greater preferential solvation of the protein at higher concentrations. This reversal is a consequence of the saturation of the cation-specific protein surface binding sites, and the different molarities of water in the bulk solutions implied by the cation sizes. These effects are mostly independent of the anion that composes the IL.

## Graphical abstract

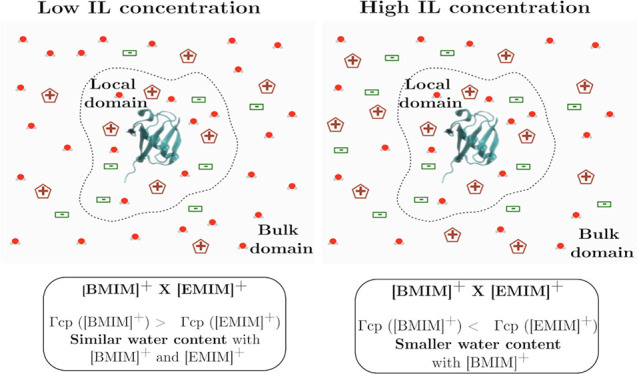

## Introduction

The solvent environment is critical for biological reactions and processes.^1^ Solvents can interact with proteins and other biomolecules through dipole–dipole, electrostatic, van der Waals, hydrogen bonding, and hydrophobic interactions.^2^ Solute–solvent interactions, particularly protein hydration, are of fundamental importance for the stability of folded structures.^3^ Understanding solvation can be complex due to the heterogeneous nature of the biomolecular surfaces, the presence of cosolvents (such as osmolytes), the intricacies of tertiary structures, and the various types of interactions that the solvent or cosolvent may establish with the proteins.^4–11^ In this context, Ionic liquids (ILs), composed mainly of organic cations and organic or inorganic anions,^12–14^ have gained significant attention due to their ability to establish a wide range of interactions with biomolecules.^15–19^ The unique solvation properties of ILs originate from the asymmetry and charge delocalization of the ions, and the possible presence of nonpolar groups, such as aliphatic side chains.^20^ As such, ILs have emerged as promising media in biotechnology,^19^ where the stabilization,^21^ activity modulation,^22^ or selective precipitation of proteins in nonconventional solvents is desired.^23^ ILs can improve the operational stability of enzymes under extreme conditions, enhance their solubility and reusability, and offer tunable environments for protein folding and unfolding processes.^24^ These applications demand a molecular-level understanding of how ILs interact with protein surfaces, particularly under aqueous conditions relevant to biological functionality.^25^

ILs can interact with proteins through hydrogen bonds, established typically by the anions, Coulombic interactions with charged residues, and dispersive-like interactions.^26,27^ ILs cations, typically of greater hydrophobic character, might alter the chemical environment surrounding the proteins by establishing dispersive-like interactions with residues in the protein surface.^28^ The surrounding chemical environment of the protein can also be perturbed by anions breaking the hydrogen bonds between the protein and water.^29,30^ In parallel, a known stabilization effect of ILs on protein stability comes from the increased viscosity of the solutions, which slows down the protein motions. The viscosity can also be tuned by increasing the size of the ions.^31,32^

The interaction of ILs with polar or apolar groups can be modulated by the choice of cation and anion.^19,33^ Ions with long alkyl chains (most commonly found in the cations) increase the hydrophobicity of the ionic liquid. The composition of the IL is also crucial to modulate its aqueous solubility and its competition with water for the interactions with the protein.^28,34^ Water can form hydrogen bonds with most common anions; however, it is typically not as effective at solvating the typically more hydrophobic cations.^7,34–36^ Water molecules can effectively solvate the anions, while the interactions with the cations are of greater complexity, and become less favorable with the increase of the cation alkyl chain.^37–39^ Aromatic cations, like those present in imidazolium and pyridinium-based ILs, typically exhibit greater water solubility compared to aliphatic cations, which can be seen in pyrrolidinium and piperidinium-based ILs.^40^ The balance between water–protein, water-IL, and IL-protein interactions determines if the net effect of the aqueous IL solution is to denature or protect the protein structure.^32,35^

We have recently investigated the structure and thermodynamics of Ubiquitin in aqueous IL solutions, focusing on the cooperative solvation of the protein by cations and anions,^9^ competitive anion effects,^9,41^ and the role of solvation in protecting or denaturing Ubiquitin in various folding states.^42^ We have shown that specific anion-protein interactions play a critical role in solvation, but that the solvation effects are not simply additive when multiple anions are present. Additionally, ILs can be found to be preferentially bound or excluded from the protein folded states, but denatured states, exposing the protein hydrophobic core, interact strongly with the IL cations, thus favoring denaturation.

A recent study by Shrivastava and co-workers^43^ has provided additional experimental evidence regarding the influence of aqueous imidazolium-based ILs on the thermodynamic and kinetic stabilities of ubiquitin. Employing techniques such as CD spectroscopy, NMR, and single-molecule force spectroscopy (SMFS), they demonstrated that hydrophobic interactions are key factors driving ubiquitin destabilization in ILs. Their findings showed that at IL concentrations of at most 1.0 mol L^–1^, cations with longer alkyl chains, such as [BMIM]^+^, exert a stronger destabilizing effect compared to shorter-chain cations like [EMIM]^+^, highlighting the critical role of cation hydrophobicity. At higher IL concentrations, more complex behaviors may emerge, potentially altering the observed effects. While such conditions could pose significant experimental challenges, they remain entirely accessible to computational approaches, offering an opportunity to explore these systems in greater detail.

Here, we employ molecular dynamics simulations to investigate how the cation’s alkyl chain length, and thus the cation hydrophobicity, affects the solvation structure of Ubiquitin by ionic liquids. Specifically, we compare the interactions of the protein with two cations: 1-butyl-3-methylimidazolium ([BMIM]^+^) and 1-ethyl-3-methylimidazolium ([EMIM]^+^). Our analysis focuses on the differences in how these ILs interact with the protein surface at varying concentrations, emphasizing the role of increased cation hydrophobicity in driving water exclusion, promoting solvent aggregation, and influencing preferential solvation. We show that the increased hydrophobicity of [BMIM]^+^ enhances its accumulation near the protein surface, especially in systems with strongly interacting anions like [DCA]^−^ and [NO_3_]^−^. At low IL concentrations (≤1.5 mol L^–1^), [BMIM]^+^-based ILs exhibit stronger preferential solvation compared to [EMIM]^+^. However, at higher concentrations (≥2.5 mol L^–1^), the protein surface sites that differentiate the cations become saturated. The relative preferential solvation is then determined by the properties of the bulk solution, particularly by the lower water molarity of solutions with [BMIM]^+^. These findings provide insights into the interplay between IL composition and protein hydration, with implications for understanding protein stability and design in IL-rich environments.

## Methods

We investigated the solvation of Ubiquitin (PDB id. 1UBQ^44^) in eight different ILs. These ILs were composed of either 1-ethyl-3-methylimidazolium ([EMIM]^+^) or 1-butyl-3-methylimidazolium ([BMIM]^+^) cations, paired with one of four anions: dicyanamide ([DCA]^−^), nitrate ([NO_3_]^−^), tetrafluorborate ([BF_4_]^−^), or chloride ([Cl]^−^). Figure 1 provides the molecular structures of all ions used. Since Ubiquitin does not possess a net charge, adding counterions for neutralization was unnecessary. Simulations were conducted at total ionic liquid reference concentrations (C_IL_) of 0.5, 1.0, 1.5, 2.0, 2.5, and 3.0 mol L^–1^.

**Figure 1:**
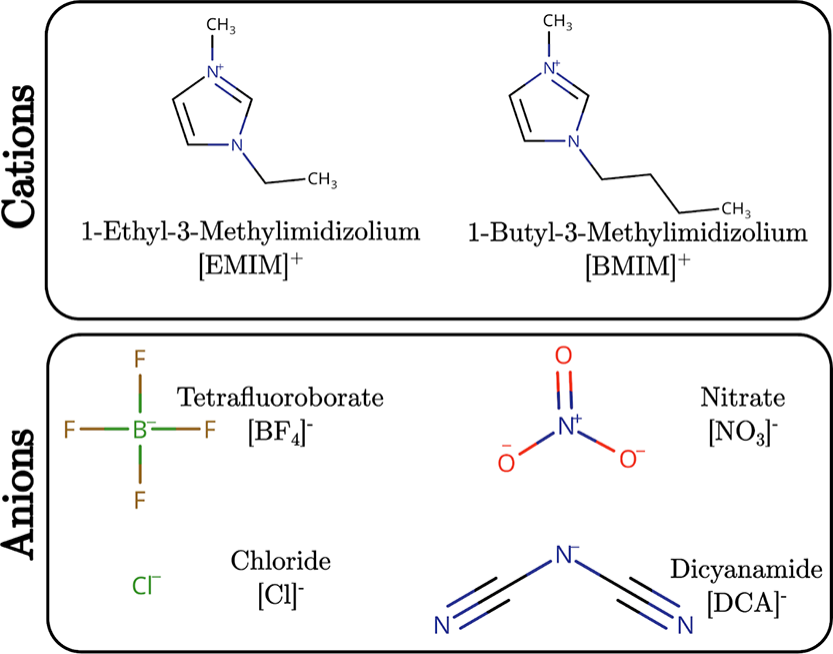
Molecular structures of the ions studied as components of the aqueous ionic liquids.

Equilibrium molecular dynamics simulations were performed using the GROMACS 2023.3 software.^45,46^ The ILs parameters were described using the virtual-site OPLS force fields,^47^ while the protein was modeled with the OPLS-AA force fields.^48^ The TIP3P model was used for water molecules.^49^ Numerical integration of the equations of motion was executed using the Verlet-leapfrog algorithm with a time step of 2 fs. A cutoff of 1.0 nm was employed for short-range electrostatic and Lennard-Jones interactions. Long-range electrostatic interactions were computed using the particle-mesh Ewald sum (PME) method,^50^ featuring a fourth-order interpolation and a grid spacing of 0.16 nm. The simulation temperature was maintained at 300 K using the modified Berendsen thermostat with a relaxation time of 0.1 ps.^51,52^ The Parrinello–Rahman algorithm was utilized to keep the pressure constant at 1 bar, with a relaxation time of 2 ps and an isothermal compressibility of 4.5 × 10^–5^ bar.^53,54^

The simulations were initiated from the crystallographic structure of ubiquitin. For each system, 20 independent boxes were generated using packmol,^55,56^ with randomly distributed solvent molecules around the protein matching the desired concentration (e.g., 0.5 mol L^–1^ of IL), ensuring variation in solvent configurations while maintaining the starting protein conformation. Each system was initially subjected to energy minimization comprising 50,000 Steepest-Descent steps with fixed protein coordinates. This was followed by thermal equilibration in the NVT ensemble for 1 ns, and then 5 ns of molecular dynamics under isothermal–isobaric (NPT) conditions, with the protein backbone constrained by applying harmonic constraints with a 10 kJ mol^–1^ Å^–2^ force constant to the CA atoms. Structural restrictions were then lifted, and 1 ns simulations were conducted under constant pressure and temperature, followed by production simulations for 10 ns also in the NPT ensemble. To ensure adequate sampling of the solvent, 20 independent simulations were performed for each system.^9,42,57^ We opted for multiple production simulations of 10 ns because the purpose of the study is to understand the solvation of the native state of Ubiquitin in various solvents.

Distributions of RMSDs and solvent accessible surface areas^58^ were calculated. SASAs for all systems support the fact that all simulations sampled similar structures, which are fluctuations around the folded model (Supplementary Figures S18–S21). Specifically, the SASA values remained within a narrow range of 48 to 50 nm^2^ across all conditions, while RMSD values fluctuated between 1 and 3 Å, indicating native-like dynamics. These consistent distributions demonstrate that the protein retains its overall structural integrity regardless of the solvent environment in the time scale of these simulations.

The ComplexMixtures.jl^59^ package was used to compute minimum-distance distribution functions (MDDFs),^60^ their decompositions into contributions of residue-types and density maps, and the Kirkwood-Buff (KB) integrals and associated preferential interaction parameters.^9,42,57^ The density of solvent molecules at each distance was obtained by histogramming the average number of minimum-distances at each 0.1 Å bin.

MDDFs provide a clear picture of solvation because the minimum-distance count is insensitive to the complexity of the solute and solvent shapes.^61^ The Kirkwood-Buff integrals, *G*_*cp*_(*R*) between the protein, *p*, and a solvent component, *c*, were calculated up to a finite distance *R* with eq 1:

$$G_{cp}\left(R\right)=\frac{1}{\rho_{c}}\left[N_{cp}\left(R\right)-N_{cp}^{*}\left(R\right)\right]$$ 1

where ρ_*c*_ is the bulk concentration of the solvent species in the solution, *N*_*cp*_(*R*) is the number of solute–solvent minimum distances smaller than *R* in the simulation, and *N*_cp_^*^(*R*) is the minimum-distance count in a reference state without solute–solvent interactions but with the same density of the bulk solvent.^59,60^ *G*_*cp*_(*R*) converges when *R* is large enough such that the presence of the solute does not affect the distribution of the solvent molecules. The reference state configurations are generated by the ComplexMixtures.jl package for each frame by computing random positions for solvent molecules with conformations sampled from the bulk phase of the simulation, with a density corresponding to the bulk density. The minimum-distance count is, then, repeated for these reference configurations. Formal details of the procedure are described in previous publications.^59,60^ Each system’s effective concentration was recalculated from the NPT production runs using the bulk region of the simulation box.^62–64^

Despite starting from independent random solvent distributions, the MDDFs of production runs were virtually identical for all 20 replicas of each system, evidencing the equilibration of the solvation structure. Adequate convergence of MDDFs to bulk density in all systems (Supplementary Figures S1–S4) and KB integral convergence for most systems was observed with R = 20 Å, as reported in previous publications.^60,65^ The solution volume closer to the solute than this distance was considered the “protein domain”, while the volume outside this domain was used to deduce the structure and thermodynamic properties of the solution without the protein. Effective bulk concentrations of the solutions were obtained from the simulations by computing the density of each solvent in the region between 20 and 30 Å from the protein surface, thus from an open subsystem of the simulation, minimizing finite-size effects.^66^

The preferential solvation parameter (Γ) quantifies the competition between the components of the solvent for the interactions with the protein.^41,67–69^ Γ can be computed from the difference between the KB integrals of the components of the solvent using

$$\Gamma_{cp}\approx \rho_{c}\left(G_{cp}-G_{wp}\right)$$ 2

where the subscripts *cp* and *wp* refer to cosolvent–protein (the IL) and water–protein.^68–71^ If Γ_*cp*_ is positive (the KB integral of the IL is greater than that of water), the IL preferentially solvates the protein. When evaluated for water, this metric is known as the preferential hydration parameter (Γ_*wp*_). For solutes of nonzero net charge, eq 2 must be adjusted to account for ionic imbalance,^70^ but here, Ubiquitin is neutral. Finally, distance-dependent coordination numbers were computed with the *bulk_coordination* function of the MolSimToolkit.jl package v1.22.3, which uses CellListMap.jl^72^ for the fast computation of interatomic distances.

## Results and Discussion

### Cation Hydrophobicity and Local Solution Structure

The distribution functions of cations relative to Ubiquitin are shown in Figure 2, for solutions with different anions, at the reference concentration of 1 mol L^–1^. This concentration will serve as an illustrative framework for examining the hydrophobic influence of cations on protein solvation by IL ions throughout the paper. Similar results were obtained with different concentrations, and when notable differences are present, the results are discussed independently.

**Figure 2:**
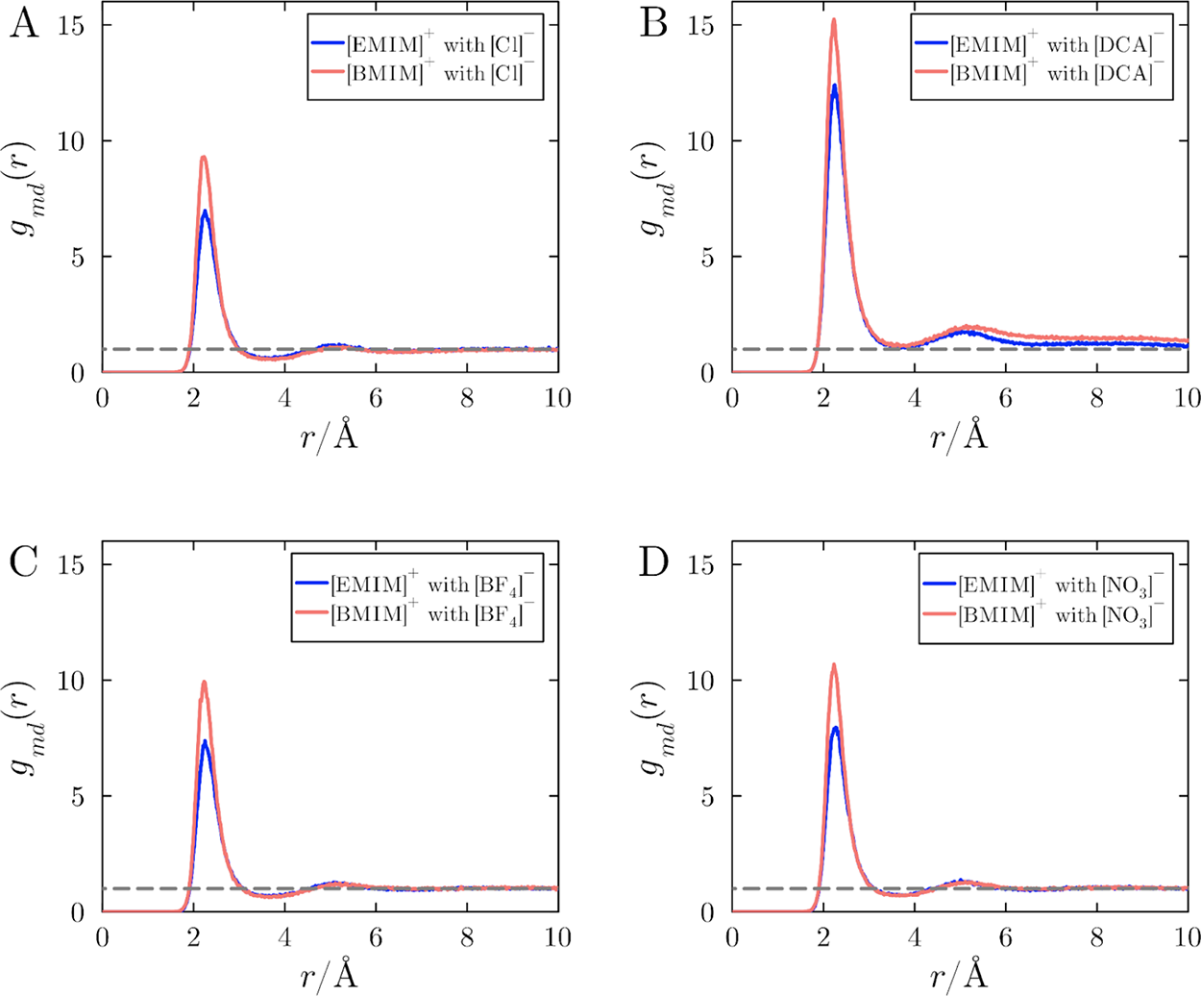
Protein-cation MDDFs for ionic liquid solutions of (A) [EMIM]­[Cl] and [BMIM]­[Cl], (B) [EMIM]­[DCA] and [BMIM]­[DCA], (C) [EMIM]­[BF_4_] and [BMIM]­[BF_4_], and (D) [EMIM]­[NO_3_] and [BMIM]­[NO_3_]. Each panel compares the MDDFs of the cations [EMIM]^+^ (blue) and [BMIM]^+^ (green) paired with various anions. The data represent mean values from 20 independent simulations at the reference concentration of 1.0 mol L^–1^. Fluctuations among replicas are negligible for this analysis.

Figure 2 shows that [BMIM]^+^ displays a greater density at the first solvation shell of Ubiquitin independently of the counterion. This greater density of [BMIM]^+^ is notably influenced by counterion correlations, which affect the distributions of both anions and cations.^9^ For instance, in Figure 2A, where the counterion is [Cl]^−^, an anion with low affinity to the protein surface, the cation density is lower than with other anions at the first peak, and the distributions at other distances are similar for [EMIM]^+^ and [BMIM]^+^. On the other hand, with the strong-binder [DCA]^−^ (Figure 2B), the first peak is greater and the density of [BMIM]^+^ is larger than that of [EMIM]^+^ at a wide range of distances. The strong interaction of [DCA]^−^ with the ubiquitin surface enhances negative charge density near the protein, driving cation accumulation on the protein surface. For the other anions (Figures 2C and 2D), the greater density of [BMIM]^+^ is noticeable only at short distances, similarly to what can be qualitatively observed for chloride.

Figure 3 contrasts the MDDFs of the anions relative to the protein, also in the 1.0 mol L^–1^ aqueous solutions of ILs with [EMIM]^+^ or [BMIM]^+^. The MDDFs exhibit common characteristics observed in recent studies:^9,41,42^ The anions form localized interactions with the protein surface atoms. For the case of [Cl]^−^, in Figure 3A, the first peak is smaller in comparison to the other anions and displaced to larger distances, and corresponds to ionic interactions of Cl^–^ ions with basic residues.^41^ The most distinct of all interactions are hydrogen bonds, which in Figure 3B-D are observed as sharp peaks at ∼ 1.9 Å.^7,35^ Among the anions, [DCA]^−^ and [NO_3_]^−^ exhibit the greatest interactions with the protein surface via hydrogen bonding.^9,41,42^

**Figure 3:**
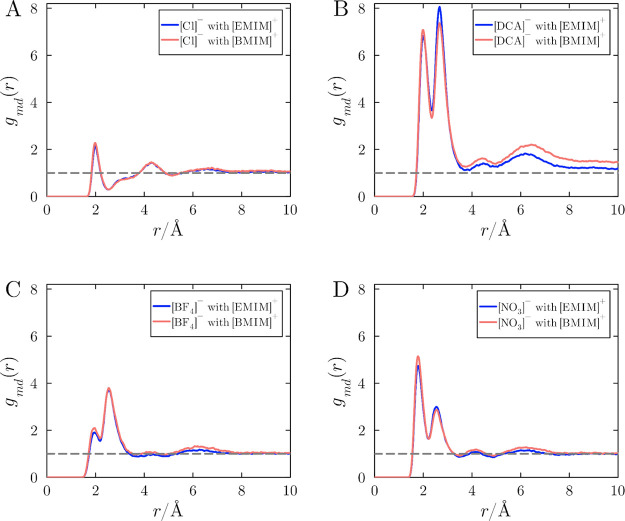
Protein-anion MDDFs for IL solutions of (A) [EMIM]­[Cl] and [BMIM]­[Cl], (B) [EMIM]­[DCA] and [BMIM]­[DCA], (C) [EMIM]­[BF_4_] and [BMIM]­[BF_4_], and (D) [EMIM]­[NO_3_] and [BMIM]­[NO_3_]. Each panel shows a comparative analysis of the anion distributions near the protein surface when paired with [EMIM]^+^ (blue) or [BMIM]^+^ (red). The data is averaged over 20 independent simulations at an IL concentration of 1.0 mol L^–1^. Fluctuations of these distributions among replicas are negligible for this analysis.

The choice of cation does not alter the qualitative shapes of the protein-anion MDDFs, thus, the nature of the interaction between the anions and protein surface is not affected. However, the probability of finding anions near the protein is influenced by the cation: The densities of anions in Figure 3, in solutions with [BMIM]^+^, are generally greater than those with [EMIM]^+^. Notable exceptions are observed in the second peak of the distributions of [DCA]^−^ and [NO_3_]^−^. These anions are strong binders to the protein surface, implying that they can accumulate more effectively close to the protein surface and carry cations along with them due to electrostatic correlations. This neutralization effect might be more effective for a smaller cation, thus promoting additional local accumulation of the anions if the cation is smaller. In general, the greater cation alkyl chain promotes, indirectly, a greater density of anions in the vicinity of the protein at this concentration. This effect is mostly independent of the chemical nature of the anion.

Details of protein solvation by the cations were explored by breaking down the MDDFs of [EMIM]^+^ and [BMIM]^+^ into the contributions of protein residue types and identities, with the *ResidueContributions* feature of ComplexMixtures.jl. Figure 4 compares the MDDFs of [EMIM]^+^ and [BMIM]^+^, both paired with [DCA]^−^, decomposed by residue type, alongside a density map representing the contribution of each specific residue to the distribution function.^59^ The cations primarily interact with neutral residues, as shown in Figures 4A and 4B, although interactions with polar, acidic, and basic residues are also possible. Since polar residues comprise an important fraction of ubiquitin’s native conformation surface, cations are expected to be found around these regions. The cations can form ionic interactions with acidic residues, and with neutral residues because of the aliphatic chains. Interestingly, the cations also approach basic residues, but the distributions (cyan in Figure 4A-B) are shifted toward larger distances, because of the presence of intermediating anions, as previously shown.^41^ Interactions with neutral residues contribute about 32.1% of the total peak magnitude at around 2.4 Å for [EMIM]^+^ and 36% for [BMIM]^+^, because of the greater alkyl chain of the latter.

**Figure 4:**
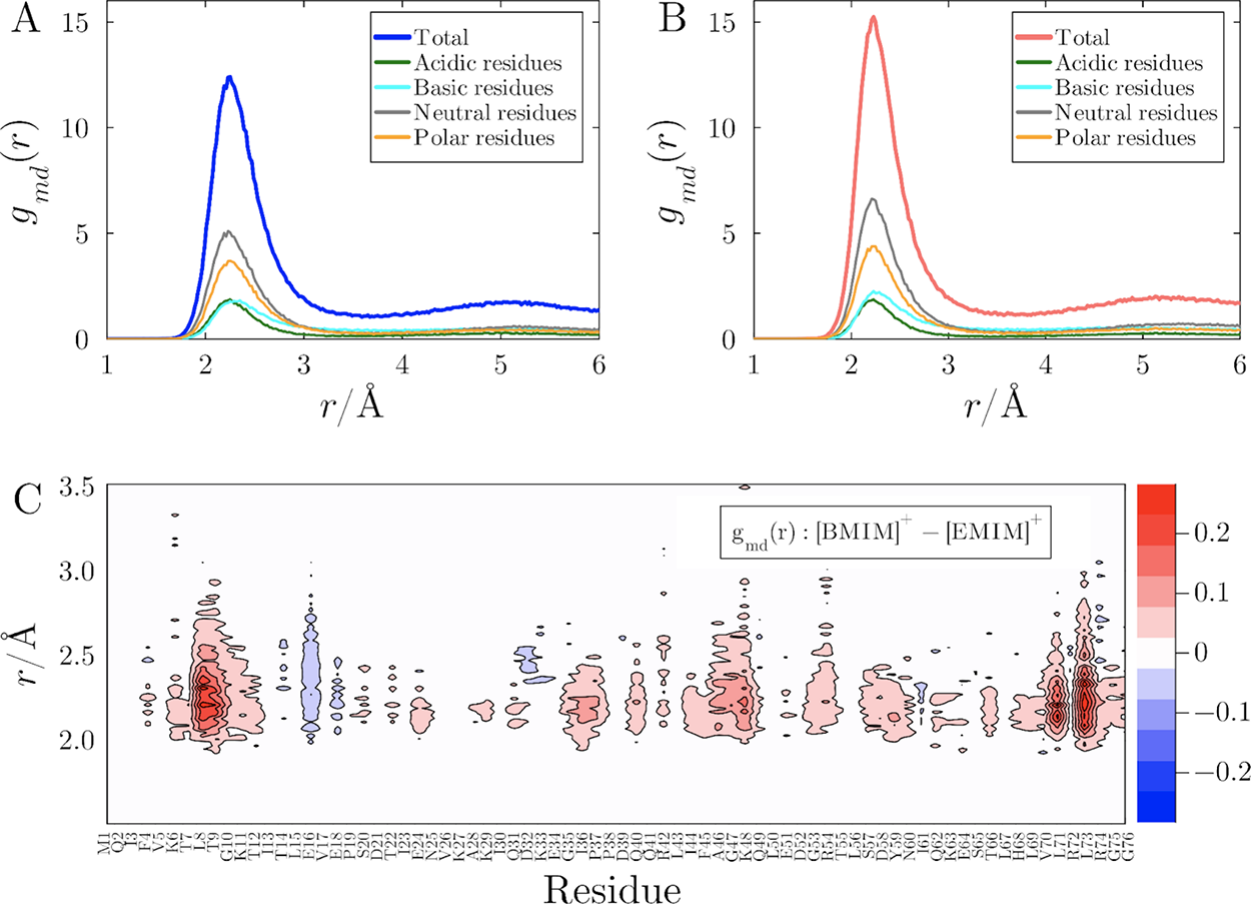
Protein-cation MDDF of (A) [EMIM]^+^ and (B) [BMIM]^+^ in solutions with [DCA]^−^ at 1.0 mol L^–1^, decomposed into contributions of basic, acidic, neutral, and polar residues. (C) Difference of residue contributions to the MDDFs of [BMIM]^+^ minus [EMIM]^+^ within 3.5 Å of the protein surface, where red indicates a greater [BMIM]^+^ local density, and blue greater [EMIM]^+^ local density. BMIM accumulation is greater around L8, L71, and L73, G47, A46, and K48. Conversely, EMIM has greater distribution than BMIM around L15, E16, and V17.

Figure 4C displays the difference in cation density, demonstrating that [BMIM]^+^ accumulates more extensively near the protein surface compared to [EMIM]^+^, particularly in hydrophobic regions and around some specific basic regions. The greater density of [BMIM]^+^ around hydrophobic residues is an expected consequence of its larger alkyl chain. The greater density around basic residues, on the other hand, must be a consequence of the greater accumulation of this cation in regions where the anion is strongly bound, associated with the greater self-association of the cations. Indeed, the cation–cation coordination number, close to the protein, is greater for [BMIM]^+^ than for [EMIM]^+^ (Supplementary Figure S11).

### Competition with Water and Preferential Solvation

The net accumulation or depletion of cosolvents around a solute can be obtained by integrating the distribution functions. Figure 5 presents the KB integrals for the anions relative to the protein paired with the two different cations at 1.0 mol L^–1^. Note that, because of the necessary electroneutrality of the solutions, the cation and anion KB integrals converge to the same values and are equivalent to the KB integrals of the complete IL (when no other ions are present and the solute is neutral). The KB integrals in Figure 5 show a greater effective accumulation of the IL in the protein domain when paired with [BMIM]^+^ relative to [EMIM]^+^. Thus, the bulkier, more hydrophobic [BMIM]^+^ cation promotes a greater IL accumulation in the protein vicinity at this concentration.

**Figure 5:**
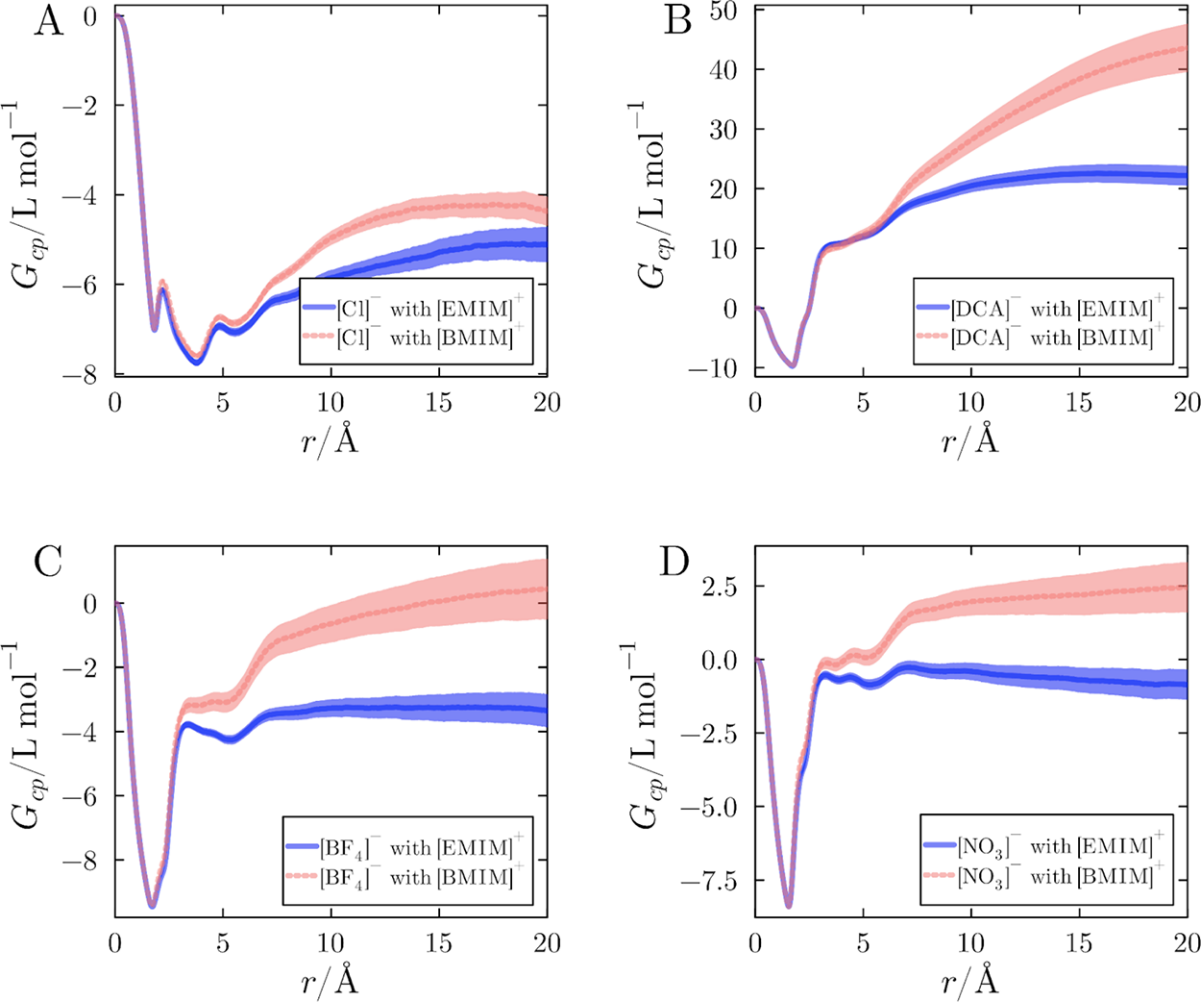
KB integrals for the anions, relative to the protein, in IL aqueous solutions with [EMIM]^+^ or [BMIM]^+^ at 1.0 mol L^–1^. (A) [Cl]^−^, (B) [DCA]^−^, (C) [BF_4_]^−^, and (D) [NO_3_]^−^. Solid lines represent the mean KB integrals averaged from 20 simulations, with shaded areas indicating the standard error of the mean.

Water–protein MDDFs and KB integrals are shown in Figure 6, at the concentration of 1.0 mol L^–1^, for IL with chloride and [DCA]^−^ (similar data for other systems can be found in Suppl. Mat. Figure S14–S17). The initial peak observed in Figure 6A, around 1.8 Å, corresponds to hydrogen bonding between water molecules and protein atoms exposed to solvent. The MDDFs for water with [EMIM]^+^ are notably greater than those with [BMIM]^+^, as shown in Figures 6A and 6C. The first peak at ∼ 1.8 Å is identical in the presence of either cation, indicating that the differences caused by the cation aliphatic tails emerge at longer distances. After the hydrogen-bonding peak, both MDDFs indicate water depletion near the protein surface; however, the depletion is more pronounced in the system containing [BMIM]^+^. This increased water exclusion can be attributed to the greater hydrophobicity of [BMIM]^+^, which accumulates more extensively near the protein at a concentration of 1.0 mol L^–1^. The KB integrals reveal a stronger exclusion of water from the protein surface in solutions containing [BMIM]^+^ compared to [EMIM]^+^, emphasizing the role of cation hydrophobicity in modulating the solvation structure, at these concentrations, consistently with recent experimental data.^43^ This effect is particularly evident when paired with dicyanamide, where water exclusion is most pronounced.

**Figure 6:**
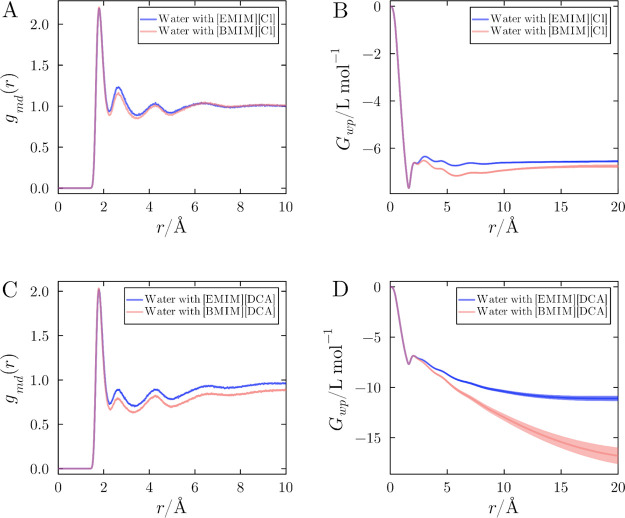
Protein hydration in ionic liquid systems containing [EMIM]­[Cl], [EMIM]­[DCA], [BMIM]­[Cl], and [BMIM]­[DCA] at 1.0 mol L^–1^. (A) Protein–water MDDF in solutions with chloride and (B) corresponding KB integrals. (C) Protein–water MDDF in solutions with [DCA]^−^ as the anion and (D) corresponding KB integrals. The KB integrals demonstrate the greater exclusion of water when in the presence of [BMIM]^+^. This underscores the stronger exclusion effect and distinct solvation structures resulting from the increased hydrophobicity of the [BMIM]^+^ cation compared to [EMIM]^+^.

### Concentration Dependence of Solvation

At low concentrations, thus, [BMIM]^+^ consistently exhibits a higher relative density near the Ubiquitin surface compared to [EMIM]^+^ independently of the accompanying anion. However, this trend shifts with changes in concentration. Figure 7 presents the MDDFs and KB integrals for IL solutions with [DCA]^−^ as the anion, at 1.0 and 3.0 mol L^–1^. At 1.0 mol L^–1^ (Figure 7A), the MDDF peak at ∼ 2.4 Å for [EMIM]^+^ is smaller than the corresponding peak for [BMIM]^+^, pointing to the fact that [BMIM]^+^ exhibits greater interactions with the protein. This is confirmed by the KB integrals in Figure 7B. However, at 3.0 mol L^–1^ (Figure 7C), this trend is reversed: The MDDF peak for [EMIM]^+^ slightly surpasses that of [BMIM]^+^ at the first solvation shell, resulting in greater KB integrals for [EMIM]^+^ solutions, shown in Figure 7D.

**Figure 7:**
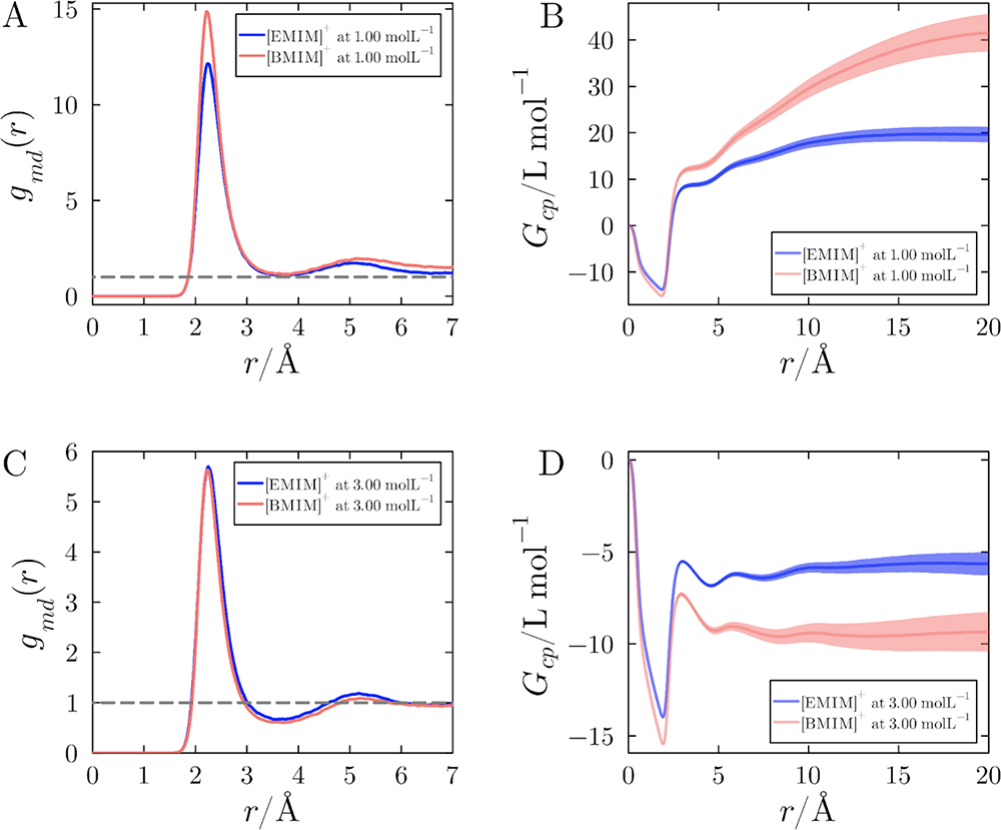
Concentration dependence of the distribution of cations relative to the protein. Protein-cation MDDFs for [EMIM]­[DCA] at reference concentrations of (A) 1.0 mol L^–1^ and (C) 3.0 mol^–1^. Corresponding Kirkwood-Buff integrals are shown in panels (B) and (D) for the same concentrations. At the greater concentration, [EMIM]^+^ becomes a stronger binder to the protein, as evidenced by slightly greater MDDFs and notably greater accumulation, as evidenced by the KB integrals.

Figure 8 shows the preferential solvation parameters (Γ_*cp*_) for all simulated ILs and concentrations. At concentrations below 1.5 mol L^–1^, Γ_*cp*_ of [BMIM]^+^ exceed those for [EMIM]^+^, particularly when paired with [DCA]^−^ and [NO_3_]^−^. Thus, at the lower concentrations, the overall picture drawn from the data in Figure 8 is that ILs with [BMIM]^+^ preferentially solvate the protein more effectively than those with [EMIM]^+^.

**Figure 8:**
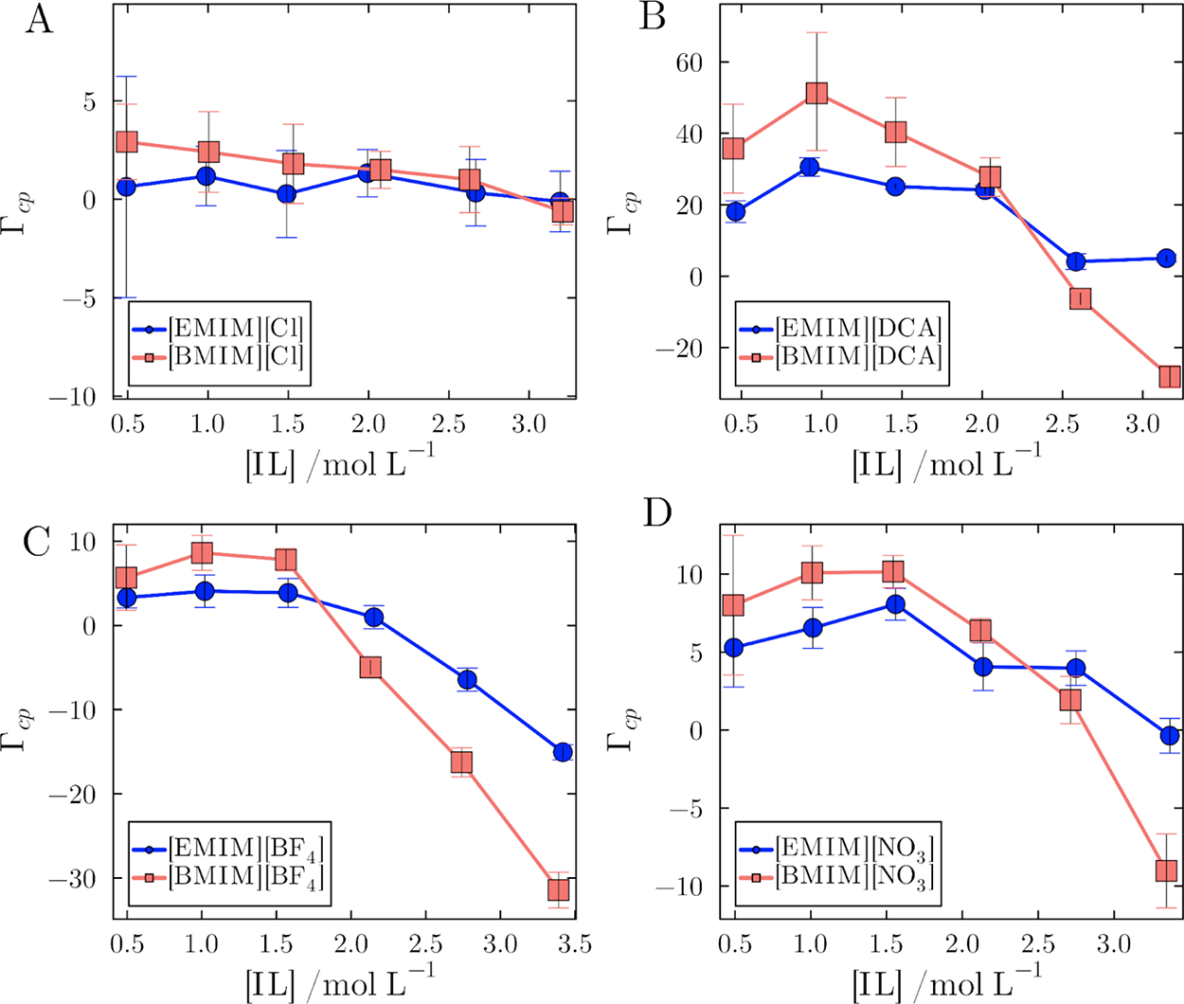
Preferential interaction parameters of the protein (Γ_cp_) of ionic liquids relative to water across different compositions and concentrations. ILs with [BMIM]^+^ are stronger binders at lower concentrations, but at higher concentrations, [EMIM]^+^ competes more favorably with water than [BMIM]^+^. (A) [EMIM]­[Cl] and [BMIM]­[Cl], (B) [EMIM]­[DCA], and [BMIM]­[DCA], (C) [EMIM]­[BF_4_] and [BMIM]­[BF_4_], and (D) [EMIM]­[NO_3_] and [BMIM]­[NO_3_]. The error bars are standard errors of the mean of 20 simulation replicates.

Interestingly, the trend is reversed at higher concentrations. After reaching a maximum Γ_*cp*_, the preferential solvation parameters decrease with concentration for all ILs. This drop is more pronounced for ILs paired with [BMIM]^+^, such that in the 2.5 and 3.0 mol L^–1^ solutions, the preferential solvation parameters for [EMIM]^+^ solutions become greater than those for [BMIM]^+^-based ILs.

A positive Γ_*cp*_ of the ILs implies protein dehydration (negative Γ_*wp*_) (supplementary data regarding Γ_*wp*_ can be found in Suppl. Inf. Tables S3 and Figure S9). At low concentrations, thus, the ILs dehydrate the protein and, to a first approximation, act as protein denaturants. However, as the concentration increases, Γ_*cp*_ decreases, and the protein becomes preferentially hydrated, most notably for ILs containing [BMIM]^+^. This would suggest, again in a first approximation, that the ILs would favor the protein folded state at higher concentrations. Note, however, that the aggregate effect of the cosolutes in protein folding depends on the interactions with the denatured states and, as we have shown previously,^42^ these ILs interact strongly with Ubiquitin denatured states, favoring denaturation at all concentrations.

### Coordination Numbers and Water Content

As shown above, at low IL concentrations, the protein is preferentially solvated by IL components, primarly driven by short-range interactions between the ions and the protein surface. Nevertheless, at higher IL concentrations, water molecules become more abundant around the protein than expected for an ideal mixture, leading to an apparent preference for hydration. Additionally, at these higher concentrations, an inversion in the solvation strength of [EMIM]^+^ and [BMIM]^+^ is observed. These concentration-dependent phenomena are further analyzed through the coordination numbers of cations and the local water concentration in each system.

Figure 9 shows that the protein-ion coordination numbers of [EMIM]^+^ and [BMIM]^+^ vary differently with concentration, in the first solvation shell. We illustrate these data for the systems in which the concentration effects are greater on the preferential solvation: the solutions with [DCA]^−^ and [BF_4_]^−^. At the lower concentrations, [BMIM]^+^ associates with the protein in greater numbers compared to [EMIM]^+^, and this is part of the explanation of the greater preferential interaction parameters and greater dehydration. Note that the greater coordination number of BMIM in the first solvation shell occurs despite its greater volume, indicating that the surface is not crowded by cations.

**Figure 9:**
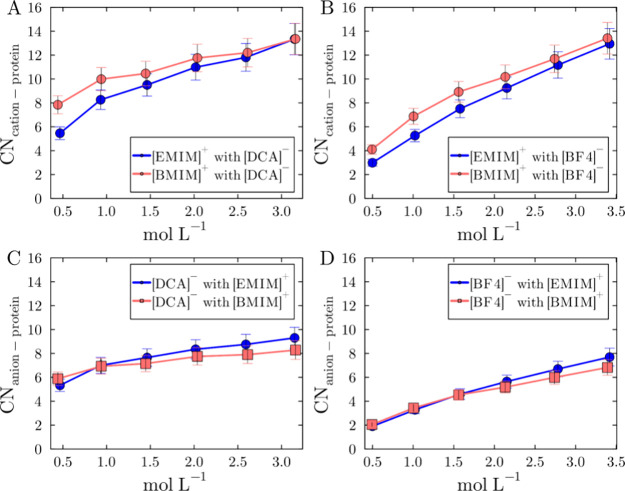
Ion-protein coordination numbers at the first solvation shell for each species, as a function of the IL concentration. (A, B) Cation–protein coordination numbers at 3.5 Å, in solutions with [DCA]^−^ and [BF_4_]^−^. (C, D) Anion-protein coordination numbers at 2.5 Å in solutions where the anions are [DCA]^−^ and [BF_4_]^−^, respectively. Error bars represent the standard errors of the mean of all simulation replicas. Coordination numbers between solvent molecules and ions in the simulated systems are provided in the Supplementary Figures S10–S12.

The differences in cation coordination numbers diminish with increasing concentration, and at approximately 2 mol L^–1^, the number of [EMIM]^+^ and [BMIM]^+^ ions in the first solvation shell becomes similar, within the observed fluctuations. The coordination numbers for anions in the first solvation shell (up to 2.5 Å - Figures 9C and 9D) also reveal a subtle trend that anions exhibit greater coordination numbers with [BMIM]^+^ at lower concentrations and the opposite at higher concentrations.

Figure 10 illustrates the distance-dependent coordination number of cations up to 5 Å from the protein surface, for the systems with [DCA]^−^, at all concentrations. The coordination numbers are zero in the protein immediate vicinity due to the exclusion volume. Beyond this region, the coordination numbers increase. At lower concentrations (Figures 10A-C), the coordination numbers of [BMIM]^+^ are greater than those of [EMIM]^+^, which complies with the greater affinity of the more hydrophobic cation to the protein surface. As the concentration increases (Figures 10D-F), the two curves become similar, indicating saturation of interaction sites that display specificity for the cations. The number of [BMIM]^+^ cations is slightly smaller at these distances, and does not seem to be the determining factor leading to the greater [EMIM]^+^ preferential solvation at the higher concentrations. In parallel, at low concentrations, there is more water in the coordination shell of the protein in the presence of [EMIM]^+^, but the difference decreases at higher concentrations (Supplementary Figure S13). Summing up, at high concentrations, the number of cations and water molecules becomes progressively more similar in the coordination shell of the protein, such that local interactions do not explain the inversion in the preferential interaction parameters.

**Figure 10:**
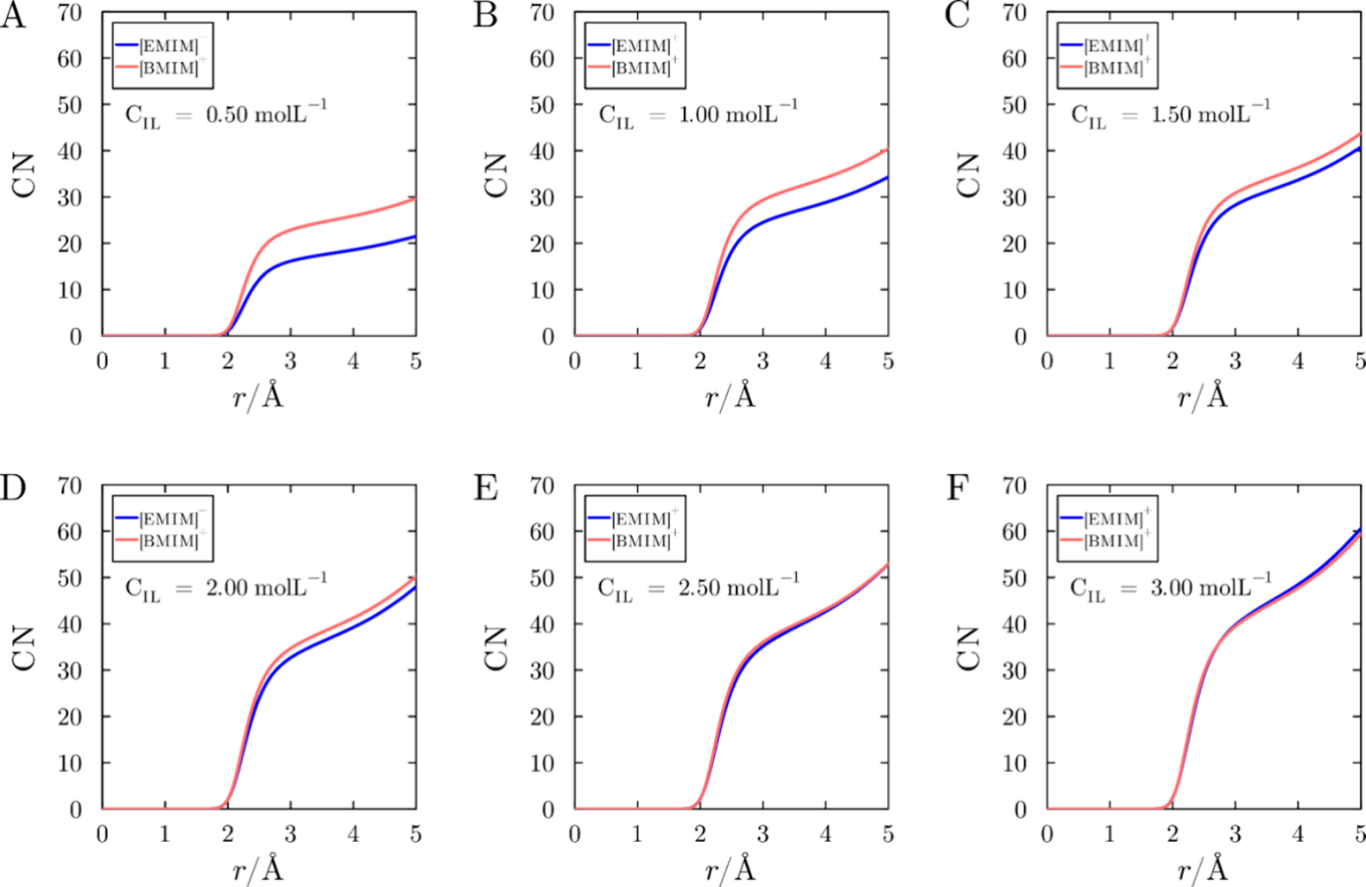
Coordination number of cations up to 5 Å from the protein surface, at increasing concentration, for the systems with [EMIM]­[DCA] or [BMIM]­[DCA]. Panels (A) to (F) display the data across IL reference concentrations, *C*_IL_ ranging from 0.5 to 3.0 mol L^–1^.

From the above analysis, it follows that the observed preferential solvation is explained by the water concentration in the bulk solutions. Table 1 displays the bulk concentrations of all chemical species in all systems for [EMIM]­[DCA] and [BMIM]­[DCA]. In the systems at 0.5 mol L^–1^, the water concentration is independent of the cation, but at higher IL concentrations, the water content drops significantly, at 3.0 mol L^–1^ from approximately 27 mol L^–1^ with [EMIM]^+^ to about 22 mol L^–1^ with [BMIM]^+^. Therefore, the bulk water concentration is significantly lower in the systems with [BMIM]^+^ compared to [EMIM]^+^. Since the number of ions in the protein vicinity is similar for both cations at high concentrations, a lower concentration of water in the bulk implies that [BMIM]^+^-based ILs are less effective in competing with water for the interactions with the protein. This, in turn, gives rise to the lower preferential solvation by [BMIM]^+^ ILs at the greater concentrations, as depicted in Figure 8.

**Table 1:** Bulk Concentrations (mol L^–1^) of Chemical Species in Systems Containing [EMIM]­[DCA] and [BMIM]­[DCA], Prepared with Reference IL Concentrations (*C*_IL_) of 0.5 and 3.0 mol L^–1^ ^a^

***C***_**IL**_mol L^**–1**^	**[EMIM]** ^**+**^	**[BMIM]** ^**+**^	**[DCA]** ^**–**^ **with [EMIM]** ^**+**^	**[DCA]** ^**–**^ **with [BMIM]** ^**+**^	**water with [EMIM]** ^**+**^	**water with [BMIM]** ^**+**^
0.50	0.469 ± 0.003	0.444 ± 0.006	0.464 ± 0.003	0.439 ± 0.006	50.70 ± 0.02	50.09 ± 0.06
3.00	3.150 ± 0.006	3.16 ± 0.02	3.148 ± 0.007	3.16 ± 0.02	27.86 ± 0.05	22.1 ± 0.2

a Similar data for all other systems and concentrations are available in Supplementary Table S2.

In summary, the coordination numbers in the solvation shell of the protein, as presented in Figures 9, 10, and Supp. Figure S13, becomes similar for the two cations and water. This indicates saturation of short-range protein-cation binding sites. The bulkier [BMIM]^+^ implies lower water content at long distances from the protein surface for the same IL molarity, implying the observed excess numbers of water and ions in the protein domain.

## Conclusions

The comparison of the solution structure and thermodynamics of ionic liquids composed of [EMIM]^+^ or [BMIM]^+^ showed that the larger alkyl chain of [BMIM]^+^ enhances protein solvation at low concentrations through water exclusion and increased cation accumulation at the protein surface. However, at higher concentrations, [BMIM]^+^ solutions display lower preferential solvation than [EMIM]^+^, and the ILs with the smaller cation become a better apparent cosolvent. This occurs because, for both cations, the sites on the protein displaying cation specificity appear to be saturated, but the bulk solution has greater water molarity for [EMIM]^+^ based ILs.

Additionally, our results demonstrate that the nature of anion distribution around the protein is largely unaffected by the specific identity of the cation, as illustrated in Figure 3. This cation-independent behavior suggests that the protein surface imposes general structural or electrostatic constraints that govern anion organization, irrespective of the surrounding cation. Consequently, the MDDFs for a given anion remain remarkably similar across the different cation environments simulated, even if the coordination number of anions and, then, their accumulation vary with the cation, as discussed throughout the paper.

The generality of the present results for different proteins can be speculated. A typical protein-charged surface should not significantly affect the results, as the most important interactions observed for the effects discussed here are of hydrophobic nature. Proteins with very high surface charges might display qualitatively different behavior, though, because of the correlated binding of ions to the surface. The size of the proteins raises a different level of discussion: here, we discuss the solvation properties of the protein folded states only, and thus, the nature of the surface is the determining factor. If the proteins were larger or smaller, but with an average similar chemistry at the surface, the results should be similar. On the other side, if the stability was evaluated taking into account the exposure of the core of the proteins, as in a previous work,^42^ the ratio of hydrophobic and hydrophilic residues might change significantly with protein size, thus likely affecting significantly the denaturing propensity of the ILs associated with the hydrophobicity of the cation.

## Supplementary Material



## Notes

Number of components for each simulation box, including cations, anions, and water molecules (Table S1 and Figure S9); post-NPT concentrations of all components in the bulk region (Table S2); data on preferential solvation (Γ_cp_) and preferential hydration (Γ_wp_) parameters across varying IL concentrations (Table S3); minimum-distance distribution functions (MDDFs) of anions, cations, and water molecules around the protein (Figures S1–S5, S10, S13, S14, and S16); Kirkwood–Buff integrals (KBIs) of water for different IL systems (Figures S15 and S17); coordination numbers of water molecules in the bulk, calculated within 5 Å of the reference species (Figures S10–S13); density difference maps for solvent components around protein residues (Figures S6–S8); distribution of SASA and RMSD calculated for ubiquitin in all different IL solutions simulated (Figures S18–S21) (PDF)
