# Supplementary material for: Cation Hydrophobicity Effects on Protein Solvation in Aqueous Ionic Liquids
Source: J Phys Chem B. 2025 May 29;129(27):6765–76. doi: 10.1021/acs.jpcb.5c00779 (PMC12257532; doi:10.1021/acs.jpcb.5c00779)
Supplement: Supplementary file 1 [file jp5c00779_si_001.pdf]

Supporting Information

Cation Hydrophobicity Effects on Protein Solvation in Aqueous Ionic Liquids

Vinicius Piccoli[1] and Leandro Martínez[1,]*

Institute of Chemistry and Center for Computing in Engineering & Science, Universidade Estadual de Campinas (UNICAMP), 13083-861 Campinas, SP, Brazil

---

Corresponding Author:

*Leandro Martínez: lmartine@unicamp.br, Institute of Chemistry, Universidade Estadual de Campinas (UNICAMP). 13083-970, Campinas, SP. Brazil. http://m3g.iqm.unicamp.br

Contents:

1) Number of components for each simulation box, including cations, anions, and water molecules (Table S1 and Figure S9).
2) Post-NPT concentrations of all components in the bulk region (Table S2).
3) Data on preferential solvation (Γ_{cp}) and preferential hydration (Γ_{wp}) parameters across varying IL concentrations (Table S3).
4) Minimum-distance distribution functions (MDDFs) of anions, cations, and water molecules around the protein (Figures S1–S5, S10, S13, S14, S16).
5) Kirkwood-Buff integrals (KBIs) of water for different IL systems (Figures S15, S17).
6) Coordination numbers of water molecules in the bulk, calculated within 5 Å of the reference species (Figures S10–S12, S13).
7) Density difference maps for solvent components around protein residues (Figures S6–S8).
8) Distribution of SASA and RMSD calculated for ubiquitin in all different IL solutions simulated (Figures S18-S21).

Information on the Initial Boxes

Table S1 – Reference concentrations (**RC**), in mol L^{-1}, are the target initial concentrations for the system setup. **Box sides** represent the dimensions of the cubic box after equilibration, where all boxes initially had a starting length of 95 Å. Number of cations (**Cation**), anions (**Anion**), and water molecules (**Water**) in each system are shown.

IL	RC (mol/L)	Box sides (Å)	Cation	Anion	Water
[EMIMCl]	0.5	93.730 ± 0.001	241	241	25202
[EMIMCl]	1.0	93.610 ± 0.001	482	482	23553
[EMIMCl]	1.5	93.481 ± 0.001	723	723	21904
[EMIMCl]	2.0	93.342 ± 0.000	965	965	20248
[EMIMCl]	2.5	91.818 ± 0.001	1226	1226	17316
[EMIMCl]	3.0	91.975 ± 0.199	1471	1471	15320
[BMIMCl]	0.5	93.948 ± 0.001	245	245	24925
[BMIMCl]	1.0	93.522 ± 0.001	490	490	22548
[BMIMCl]	1.5	93.079 ± 0.001	736	736	20160
[BMIMCl]	2.0	92.629 ± 0.001	981	981	17783
[BMIMCl]	2.5	92.177 ± 0.000	1226	1226	15405

IL	RC (mol/L)	Box sides (Å)	Cation	Anion	Water
[BMIMCl]	3.0	91.738 ± 0.001	1471	1471	13028
[EMIMDCA]	0.5	95.051 ± 0.001	254	254	25761
[EMIMDCA]	1.0	94.685 ± 0.001	507	507	23292
[EMIMDCA]	1.5	94.322 ± 0.001	760	760	20824
[EMIMDCA]	2.0	93.975 ± 0.001	1014	1014	18357
[EMIMDCA]	2.5	93.640 ± 0.000	1269	1269	15888
[EMIMDCA]	3.0	93.288 ± 0.001	1522	1522	13420
[BMIMDCA]	0.5	95.107 ± 0.000	254	254	25353
[BMIMDCA]	1.0	94.784 ± 0.001	507	507	22475
[BMIMDCA]	1.5	94.471 ± 0.001	760	760	19599
[BMIMDCA]	2.0	94.189 ± 0.001	1014	1014	16723
[BMIMDCA]	2.5	94.036 ± 0.001	1269	1269	13936
[BMIMDCA]	3.0	93.657 ± 0.001	1522	1522	10969
[EMIMNO3]	0.5	94.403 ± 0.000	251	251	25512

IL	RC (mol/L)	Box sides (Å)	Cation	Anion	Water
[EMIMNO3]	1.0	93.731 ± 0.000	502	502	23097
[EMIMNO3]	1.5	93.047 ± 0.001	752	752	20692
[EMIMNO3]	2.0	92.346 ± 0.000	1003	1003	18277
[EMIMNO3]	2.5	91.632 ± 0.001	1254	1254	15862
[EMIMNO3]	3.0	90.902 ± 0.001	1505	1505	13447
[BMIMNO3]	0.5	94.473 ± 0.001	251	251	25123
[BMIMNO3]	1.0	93.864 ± 0.000	502	502	22319
[BMIMNO3]	1.5	93.241 ± 0.001	752	752	19527
[BMIMNO3]	2.0	92.599 ± 0.001	1003	1003	16723
[BMIMNO3]	2.5	91.947 ± 0.001	1254	1254	13920
[BMIMNO3]	3.0	91.286 ± 0.001	1505	1505	11116
[EMIMBF4]	0.5	94.713 ± 0.000	254	254	25432
[EMIMBF4]	1.0	94.010 ± 0.000	507	507	22646
[EMIMBF4]	1.5	93.285 ± 0.000	761	761	19849

IL	RC (mol/L)	Box sides (Å)	Cation	Anion	Water
[EMIMBF4]	2.0	92.544 ± 0.001	1014	1014	17063
[EMIMBF4]	2.5	91.787 ± 0.001	1269	1269	14266
[EMIMBF4]	3.0	91.001 ± 0.001	1522	1522	11480
[BMIMBF4]	0.5	94.783 ± 0.000	254	254	25037
[BMIMBF4]	1.0	94.151 ± 0.001	507	507	21858
[BMIMBF4]	1.5	93.497 ± 0.000	761	761	18666
[BMIMBF4]	2.0	92.824 ± 0.000	1014	1014	15486
[BMIMBF4]	2.5	92.144 ± 0.000	1269	1269	12295
[BMIMBF4]	3.0	91.433 ± 0.001	1522	1522	9115

Bulk Concentrations of Ions and Water

Table S2 – Average bulk concentrations (mol L⁻¹) and standard errors of the mean of 20 simulation replicas for each system, for each component. The reference concentrations (**RC**) are the target initial concentrations of the system setup.

IL	RC (mol/L)	[Cation] (mol/L)	[Anion] (mol/L)	[Water] (mol/L)
[EMIMCl]	0.5	0.490 ± 0.001	0.491 ± 0.001	51.511 ± 0.009
[EMIMCl]	1.0	0.990 ± 0.002	0.987 ± 0.002	48.306 ± 0.009
[EMIMCl]	1.5	1.488 ± 0.002	1.488 ± 0.001	45.132 ± 0.009
[EMIMCl]	2.0	1.992 ± 0.002	1.993 ± 0.002	41.922 ± 0.013
[EMIMCl]	2.5	2.668 ± 0.002	2.666 ± 0.002	37.673 ± 0.009
[EMIMCl]	3.0	3.185 ± 0.022	3.205 ± 0.025	33.174 ± 0.216
[BMIMCl]	0.5	0.491 ± 0.002	0.495 ± 0.001	50.619 ± 0.013
[BMIMCl]	1.0	1.007 ± 0.002	1.004 ± 0.002	46.395 ± 0.016
[BMIMCl]	1.5	1.533 ± 0.002	1.532 ± 0.002	42.102 ± 0.016
[BMIMCl]	2.0	2.075 ± 0.002	2.073 ± 0.002	37.691 ± 0.012
[BMIMCl]	2.5	2.632 ± 0.002	2.634 ± 0.002	33.155 ± 0.016
[BMIMCl]	3.0	3.209 ± 0.003	3.212 ± 0.004	28.444 ± 0.025
[EMIMDCA]	0.5	0.469 ± 0.003	0.464 ± 0.003	50.703 ± 0.024
[EMIMDCA]	1.0	0.936 ± 0.006	0.927 ± 0.007	46.757 ± 0.054
[EMIMDCA]	1.5	1.465 ± 0.012	1.460 ± 0.013	42.256 ± 0.106
[EMIMDCA]	2.0	2.020 ± 0.010	2.015 ± 0.011	37.519 ± 0.086
[EMIMDCA]	2.5	2.592 ± 0.011	2.592 ± 0.012	32.616 ± 0.100
[EMIMDCA]	3.0	3.150 ± 0.006	3.148 ± 0.007	27.857 ± 0.053
[BMIMDCA]	0.5	0.444 ± 0.006	0.439 ± 0.006	50.095 ± 0.058

IL	RC (mol/L)	[Cation] (mol/L)	[Anion] (mol/L)	[Water] (mol/L)
[BMIMDCA]	1.0	0.942 ± 0.014	0.935 ± 0.015	44.997 ± 0.155
[BMIMDCA]	1.5	1.449 ± 0.031	1.443 ± 0.032	39.800 ± 0.335
[BMIMDCA]	2.0	2.039 ± 0.024	2.036 ± 0.025	33.662 ± 0.259
[BMIMDCA]	2.5	2.612 ± 0.015	2.611 ± 0.016	27.708 ± 0.169
[BMIMDCA]	3.0	3.159 ± 0.016	3.159 ± 0.017	22.027 ± 0.175
[EMIMNO3]	0.5	0.492 ± 0.002	0.492 ± 0.002	51.073 ± 0.014
[EMIMNO3]	1.0	1.014 ± 0.003	1.014 ± 0.004	47.272 ± 0.025
[EMIMNO3]	1.5	1.557 ± 0.003	1.559 ± 0.003	43.323 ± 0.020
[EMIMNO3]	2.0	2.138 ± 0.004	2.137 ± 0.005	39.111 ± 0.033
[EMIMNO3]	2.5	2.745 ± 0.003	2.745 ± 0.003	34.718 ± 0.024
[EMIMNO3]	3.0	3.375 ± 0.004	3.374 ± 0.004	30.160 ± 0.029
[BMIMNO3]	0.5	0.486 ± 0.002	0.488 ± 0.002	50.237 ± 0.017
[BMIMNO3]	1.0	1.008 ± 0.004	1.005 ± 0.004	45.527 ± 0.039
[BMIMNO3]	1.5	1.543 ± 0.004	1.543 ± 0.004	40.701 ± 0.037
[BMIMNO3]	2.0	2.120 ± 0.004	2.122 ± 0.004	35.506 ± 0.039
[BMIMNO3]	2.5	2.715 ± 0.004	2.713 ± 0.004	30.171 ± 0.037
[BMIMNO3]	3.0	3.346 ± 0.005	3.343 ± 0.006	24.498 ± 0.049
[EMIMBF4]	0.5	0.498 ± 0.002	0.499 ± 0.002	50.379 ± 0.014
[EMIMBF4]	1.0	1.022 ± 0.003	1.022 ± 0.003	45.891 ± 0.026
[EMIMBF4]	1.5	1.578 ± 0.003	1.577 ± 0.003	41.148 ± 0.024
[EMIMBF4]	2.0	2.152 ± 0.005	2.154 ± 0.005	36.232 ± 0.043
[EMIMBF4]	2.5	2.782 ± 0.004	2.782 ± 0.005	30.895 ± 0.036
[EMIMBF4]	3.0	3.418 ± 0.004	3.420 ± 0.005	25.507 ± 0.039

IL	RC (mol/L)	[Cation] (mol/L)	[Anion] (mol/L)	[Water] (mol/L)
[BMIMBF4]	0.5	0.492 ± 0.002	0.494 ± 0.002	49.542 ± 0.019
[BMIMBF4]	1.0	1.005 ± 0.004	1.004 ± 0.004	44.224 ± 0.040
[BMIMBF4]	1.5	1.561 ± 0.006	1.559 ± 0.006	38.486 ± 0.063
[BMIMBF4]	2.0	2.133 ± 0.007	2.134 ± 0.007	32.588 ± 0.075
[BMIMBF4]	2.5	2.740 ± 0.008	2.742 ± 0.008	26.350 ± 0.086
[BMIMBF4]	3.0	3.390 ± 0.006	3.394 ± 0.006	19.666 ± 0.063

Preferential solvation and preferential hydration parameters.

Table S3 – Preferential solvation (Γ_{cp})) and preferential hydration (Γ_{wp})), and standard error of the mean of 20 simulation replicas, for all systems simulated. **RC** is the reference concentration of each system.

IL	RC (mol/L)	Γcp	Γwp
[EMIMCl]	0.5	0.7 ± 0.2	-105 ± 28
[EMIMCl]	1.0	1.2 ± 0.4	-45 ± 19
[EMIMCl]	1.5	0.6 ± 0.5	-16 ± 12
[EMIMCl]	2.0	1.4 ± 0.5	-33 ± 12
[EMIMCl]	2.5	0.6 ± 0.4	-9 ± 7
[EMIMCl]	3.0	1.5 ± 0.8	16 ± 6
[BMIMCl]	0.5	3.0 ± 0.4	-356 ± 50
[BMIMCl]	1.0	2.3 ± 0.4	-104 ± 25
[BMIMCl]	1.5	1.7 ± 0.5	-42 ± 17
[BMIMCl]	2.0	1.5 ± 0.5	-23 ± 10
[BMIMCl]	2.5	0.7 ± 0.6	-13 ± 11
[BMIMCl]	3.0	-0.6 ± 0.6	2 ± 9
[EMIMDCA]	0.5	18 ± 1	-1866 ± 117
[EMIMDCA]	1.0	30 ± 2	-1437 ± 85
[EMIMDCA]	1.5	24 ± 3	-699 ± 83
[EMIMDCA]	2.0	24 ± 4	-441 ± 70
[EMIMDCA]	2.5	3 ± 4	-34 ± 57
[EMIMDCA]	3.0	4 ± 4	-40 ± 31
[BMIMDCA]	0.5	36.6 ± 2.0	-4085 ± 251

IL	RC (mol/L)	Γcp	Γwp
[BMIMDCA]	1.0	55.4 ± 4.5	-2625 ± 211
[BMIMDCA]	1.5	41.7 ± 5.3	-1171 ± 152
[BMIMDCA]	2.0	29.8 ± 5.8	-497 ± 97
[BMIMDCA]	2.5	-6.5 ± 7.8	65 ± 84
[BMIMDCA]	3.0	-26.5 ± 8.1	172 ± 57
[EMIMNO3]	0.5	5.3 ± 0.5	-548 ± 62
[EMIMNO3]	1.0	6.5 ± 0.6	-308 ± 27
[EMIMNO3]	1.5	8.4 ± 1.0	-240 ± 29
[EMIMNO3]	2.0	4.0 ± 1.5	-72 ± 28
[EMIMNO3]	2.5	4.9 ± 1.1	-62 ± 14
[EMIMNO3]	3.0	-1.5 ± 1.4	14 ± 13
[BMIMNO3]	0.5	8.2 ± 0.5	-855 ± 61
[BMIMNO3]	1.0	10.6 ± 1.0	-477 ± 48
[BMIMNO3]	1.5	10.3 ± 1.2	-274 ± 36
[BMIMNO3]	2.0	6.4 ± 1.2	-108 ± 21
[BMIMNO3]	2.5	1.9 ± 2.1	-20 ± 24
[BMIMNO3]	3.0	-8.7 ± 1.7	65 ± 12
[EMIMBF4]	0.5	3.3 ± 0.4	-347 ± 44
[EMIMBF4]	1.0	3.8 ± 0.6	-176 ± 30
[EMIMBF4]	1.5	4.1 ± 1.1	-104 ± 29
[EMIMBF4]	2.0	0.8 ± 1.8	-18 ± 30
[EMIMBF4]	2.5	-7.0 ± 1.9	76 ± 21
[EMIMBF4]	3.0	-15.3 ± 2.5	113 ± 18

IL	RC (mol/L)	Γcp	Γwp
[BMIMBF4]	0.5	5.8 ± 0.6	-616 ± 67
[BMIMBF4]	1.0	8.2 ± 1.1	-359 ± 52
[BMIMBF4]	1.5	8.3 ± 2.0	-204 ± 54
[BMIMBF4]	2.0	-5.8 ± 2.4	87 ± 38
[BMIMBF4]	2.5	-16.5 ± 3.5	155 ± 33
[BMIMBF4]	3.0	-31.8 ± 5.2	179 ± 29

Minimum-distance distribution functions for all ionic liquids simulated.

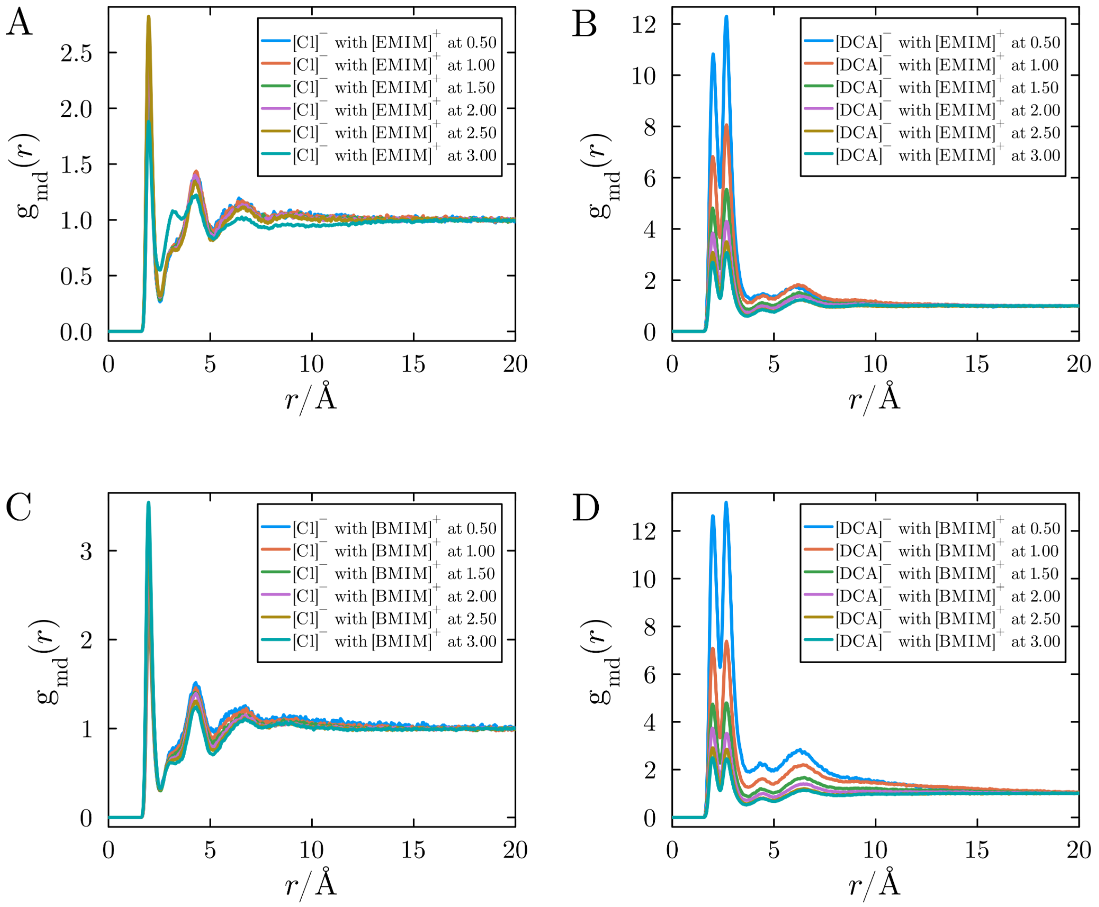

Figure S1 – Minimum-distance distribution functions (MDDFs) of anions Cl^- and DCA^- around the protein in ionic liquid (IL) solutions. The figure highlights how anions distribute with respect to the protein surface, evaluated across different reference concentrations (in mol L^{-1}). The panels illustrate MDDFs for four IL systems: **A)** [EMIM][Cl], **B)** [EMIM][DCA], **C)** [BMIM][Cl], and **D)** [BMIM][DCA]. Each curve represents the mean of 20 independent simulations.

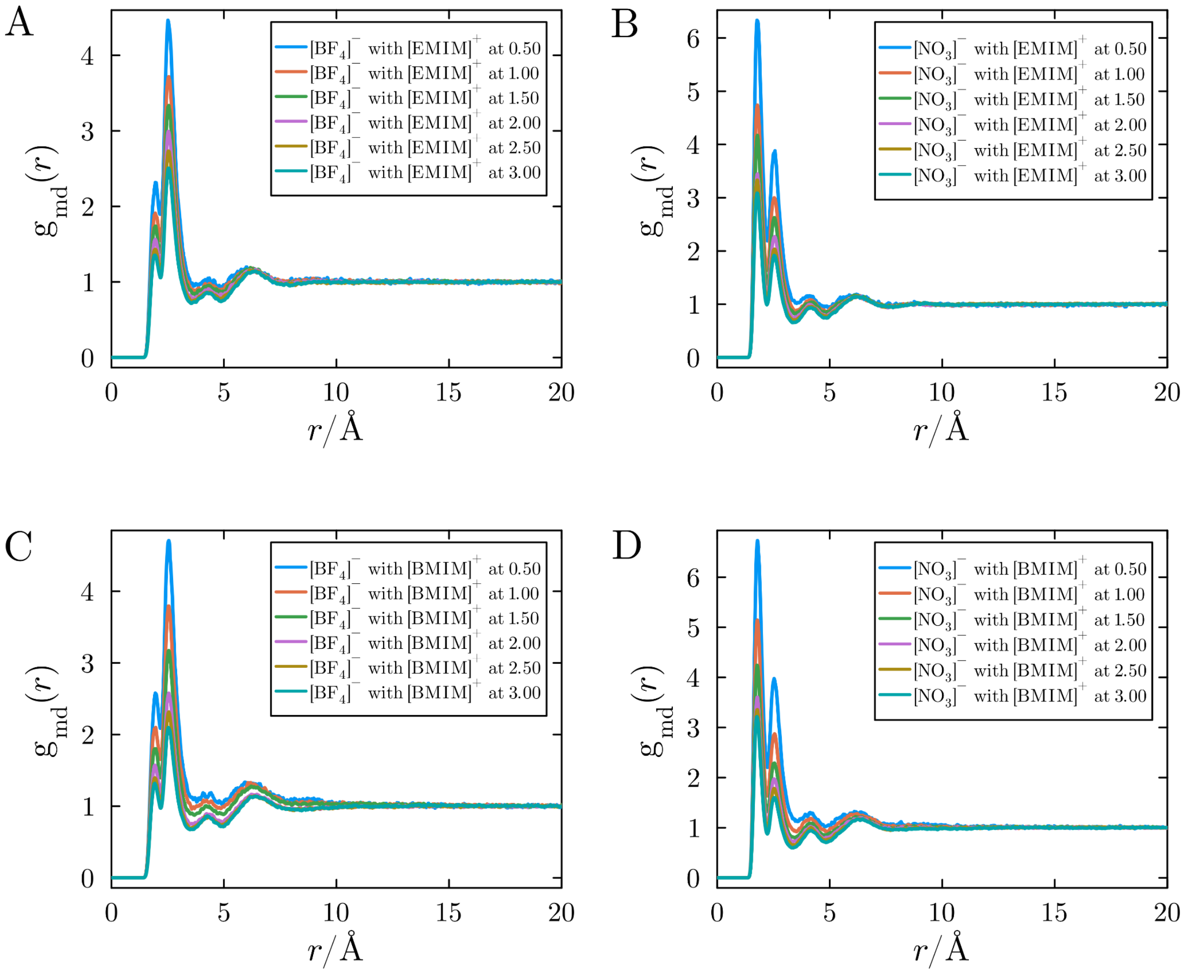

Figure S2 – Minimum-distance distribution functions (MDDFs) for the anions $[BF]_4^-$ and $[NO]_3^-$ around the protein in ionic liquid (IL) solutions. This figure illustrates the spatial distribution of anions relative to the protein surface, evaluated across a range of reference concentrations (in mol L^{-1}). The panels depict MDDFs for four IL systems: **A)** $[EMIM][BF]_4$, **B)** $[EMIM][NO]_3$, **C)** $[BMIM][BF]_4$, and **D)** $[BMIM][NO]_3$. Each curve represents the mean of 20 independent simulations, offering insights into the interaction dynamics of the anions with the protein under different ionic environments.

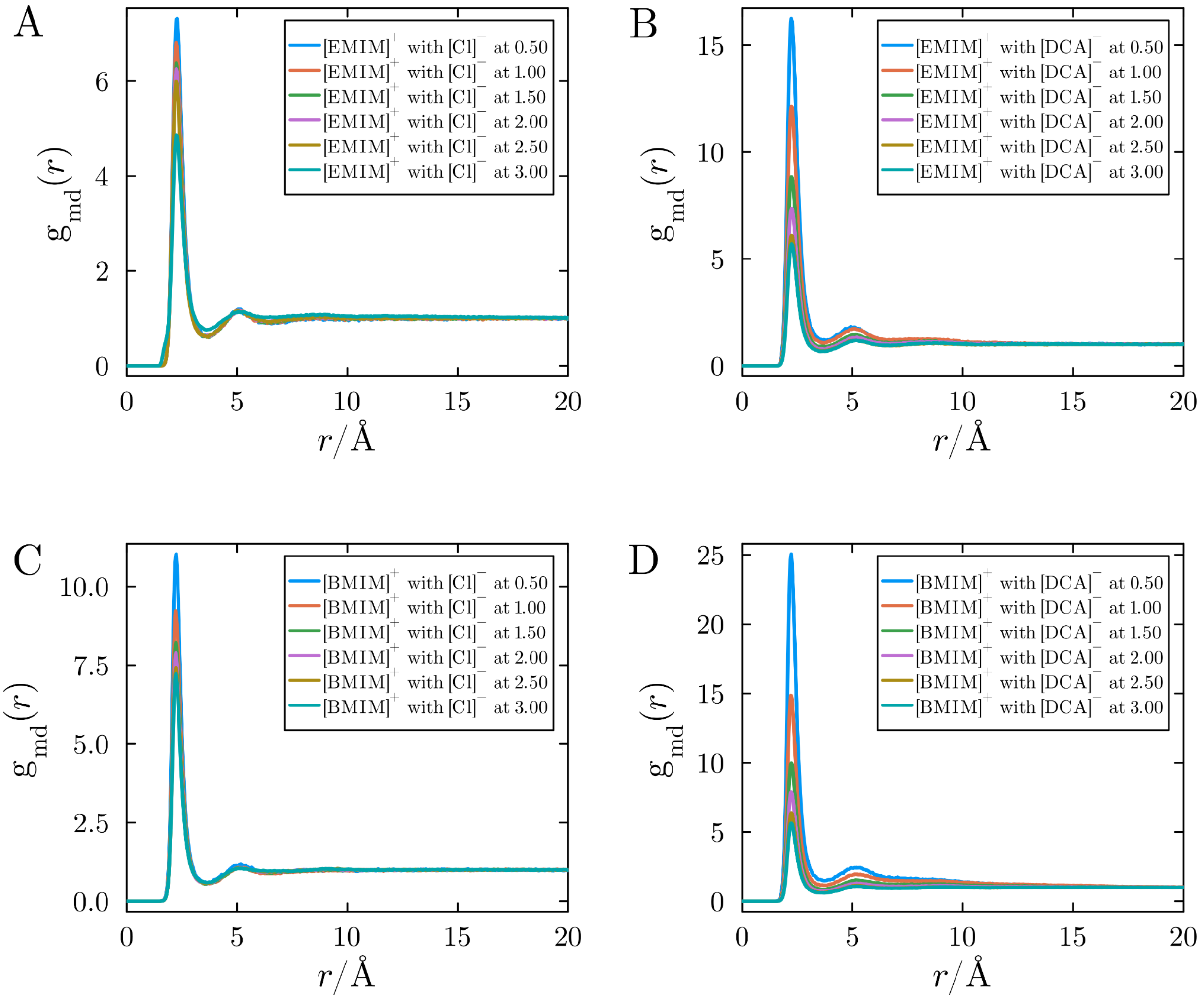

Figure S3 – Minimum-distance distribution functions (MDDFs) for the cations $[EMIM]^+$ and $[BMIM]^+$ around the protein in ionic liquid (IL) solutions, paired with the anions $[Cl]^-$ and $[DCA]^-$. This figure illustrates the spatial distribution of cations relative to the protein surface across various reference concentrations (in mol L^{-1}). The panels show MDDFs for four IL systems: **A)** [EMIM][Cl], **B)** [EMIM][DCA], **C)** [BMIM][Cl], and **D)** [BMIM][DCA]. Each curve represents the mean of 20 independent simulations, providing insights into the behavior of cations in the presence of different anions and their interaction dynamics with the protein.

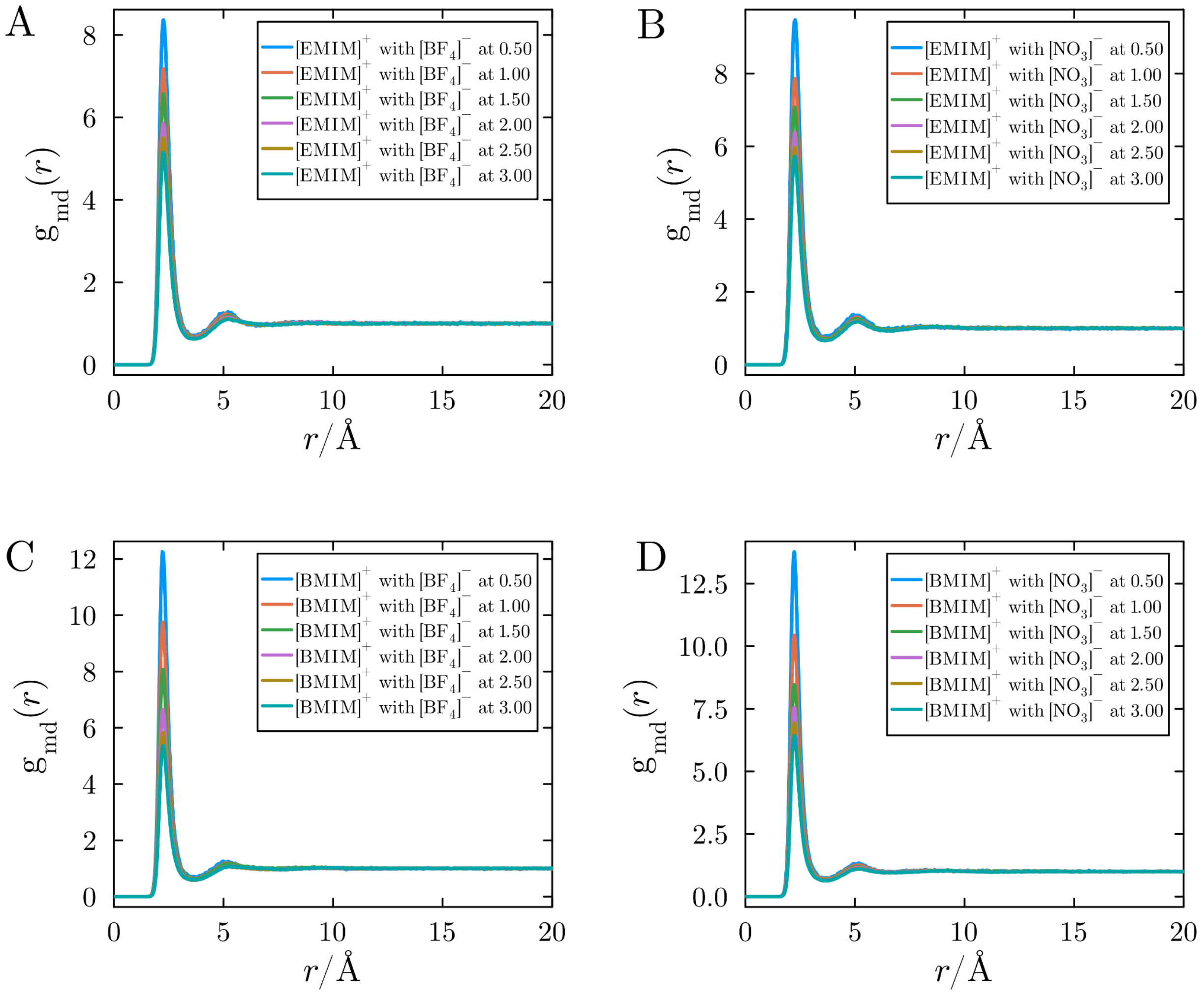

Figure S4 – Minimum-distance distribution functions (MDDFs) for the cations $[EMIM]^+$ and $[BMIM]^+$ around the protein in ionic liquid (IL) solutions, paired with the anions $[BF]_4^-$ and $[NO]_4^-$. This figure illustrates the spatial distribution of cations relative to the protein surface across a range of reference concentrations (in mol L^{-1}). The panels depict MDDFs for four IL systems: **A)** $[EMIM][BF]_4$, **B)** $[EMIM][NO]_3$, **C)** $[BMIM][BF]_4$, and **D)** $[BMIM][NO]_3$. Each curve represents the mean of 20 independent simulations, providing insights into the interaction dynamics of cations with the protein under varying ionic environments.

Density map of the ions and water around the protein residues at ~1.0 mol L^{-1}

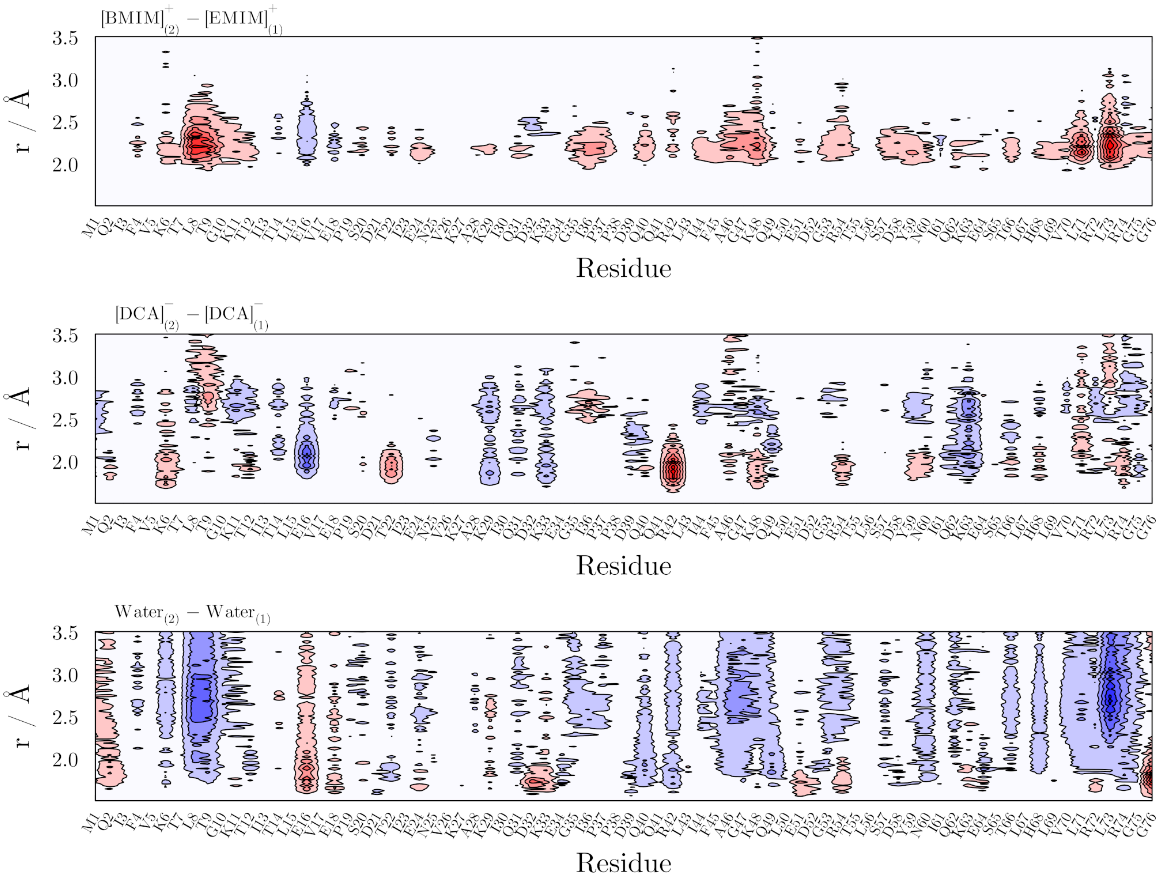

Figure S5 – Difference in protein residue contributions to the solvent-protein distribution functions for systems with [BMIM]$^+$ (denoted as underscore (2)) and [EMIM]$^+$ (denoted as underscore (1)). The density difference map compares the contributions of each protein residue to the MDDFs within 3.5 Å of the protein surface, based on data from 20 independent simulations for both cations. Red regions indicate a higher density of the solvent component in the [BMIM]$^+$ system, while blue regions indicate a higher density in the [EMIM]$^+$ system. The maps represent ~1.0 mol L^{-1} IL solutions with the anion [DCA]$^-$.

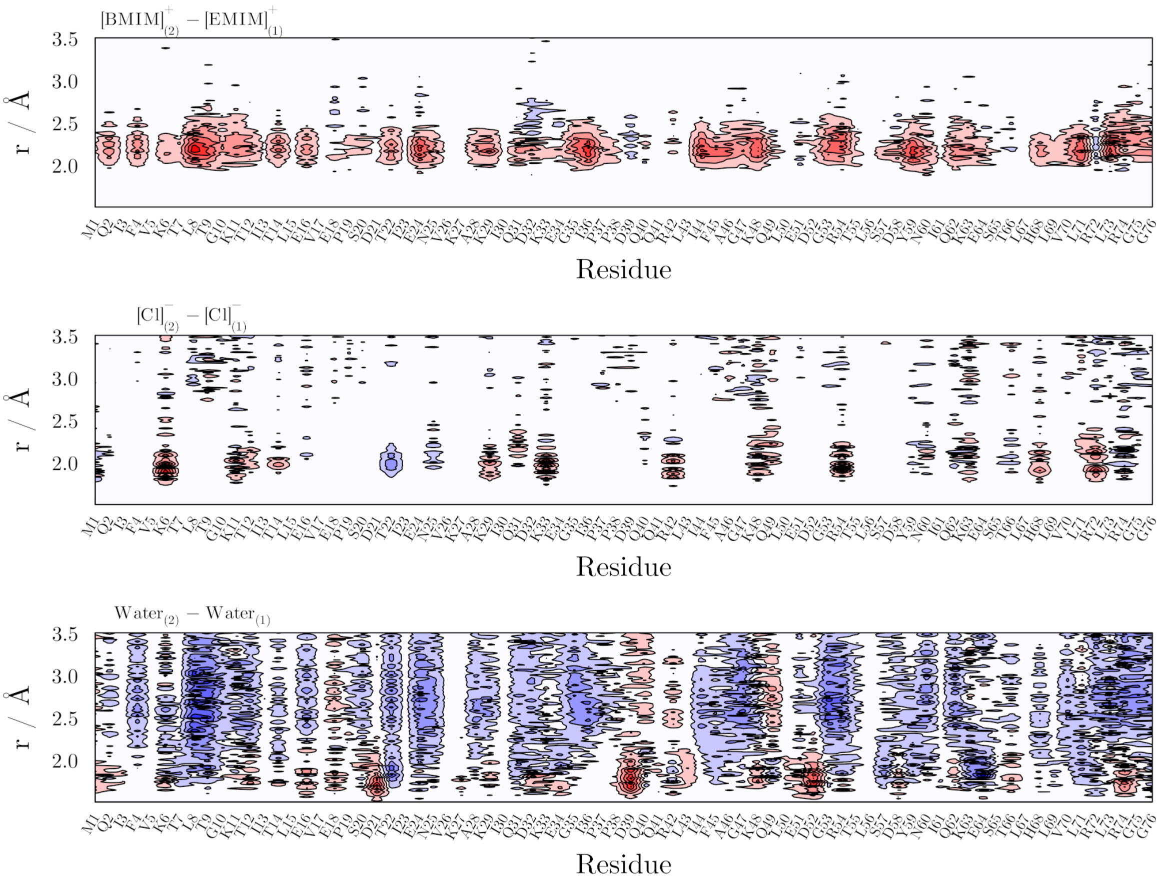

Figure S6 – Difference in protein residue contributions to the solvent-protein distribution functions for systems with $[BMIM]^+$ (denoted as underscore (2)) and $[EMIM]^+$ (denoted as underscore (1)). The density difference map compares the contributions of each protein residue to the MDDFs within 3.5 Å of the protein surface, based on data from 20 independent simulations for both cations. Red regions indicate a higher density of the solvent component in the $[BMIM]^+$ system, while blue regions indicate a higher density in the $[EMIM]^+$ system. The maps represent ~1.0 mol L^{-1} IL solutions with the anion $[Cl]^-$.

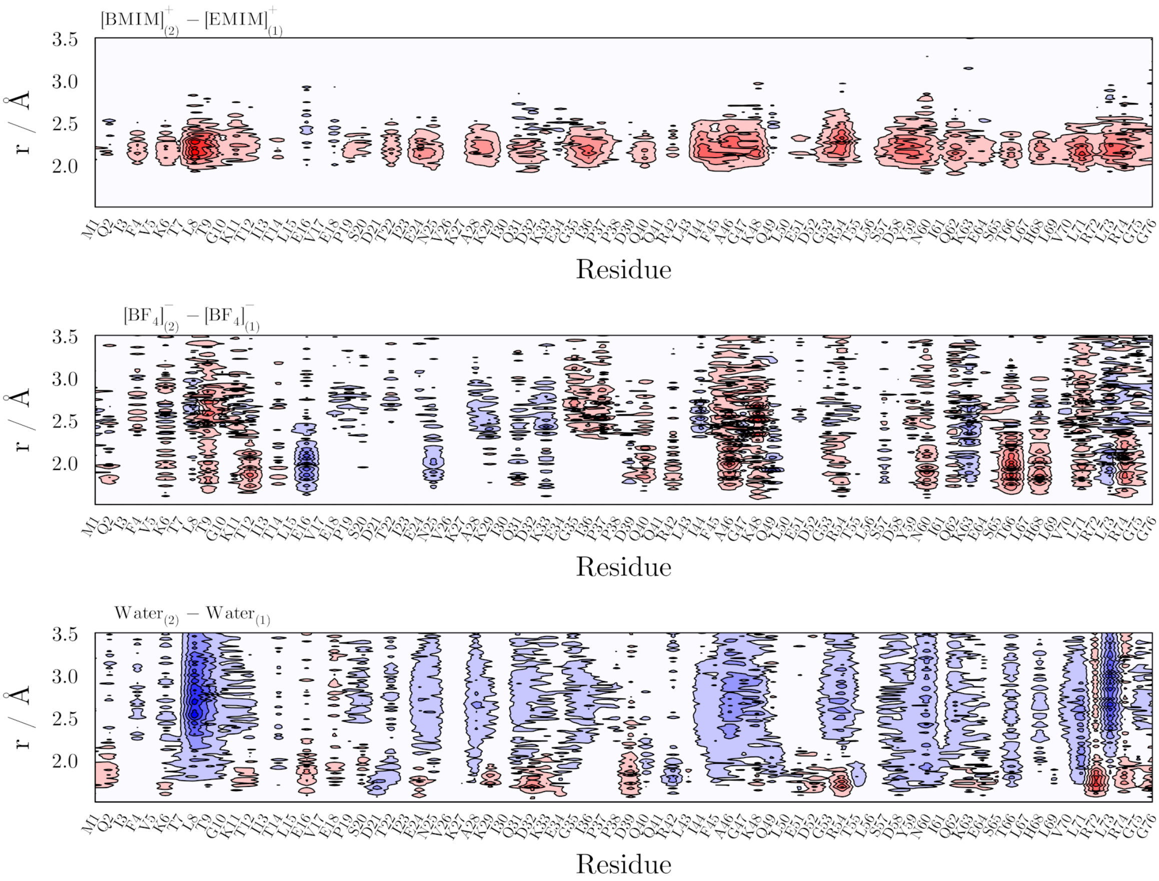

Figure S7 – Difference in protein residue contributions to the solvent-protein distribution functions for systems with [BMIM]$^+$ (denoted as underscore (2)) and [EMIM]$^+$ (denoted as underscore (1)). The density difference map compares the contributions of each protein residue to the MDDFs within 3.5 Å of the protein surface, based on data from 20 independent simulations for both cations. Red regions indicate a higher density of the solvent component in the [BMIM]$^+$ system, while blue regions indicate a higher density in the [EMIM]$^+$ system. The maps represent ~1.0 mol L^{-1} IL solutions with the anion [BF]$_4^-$.

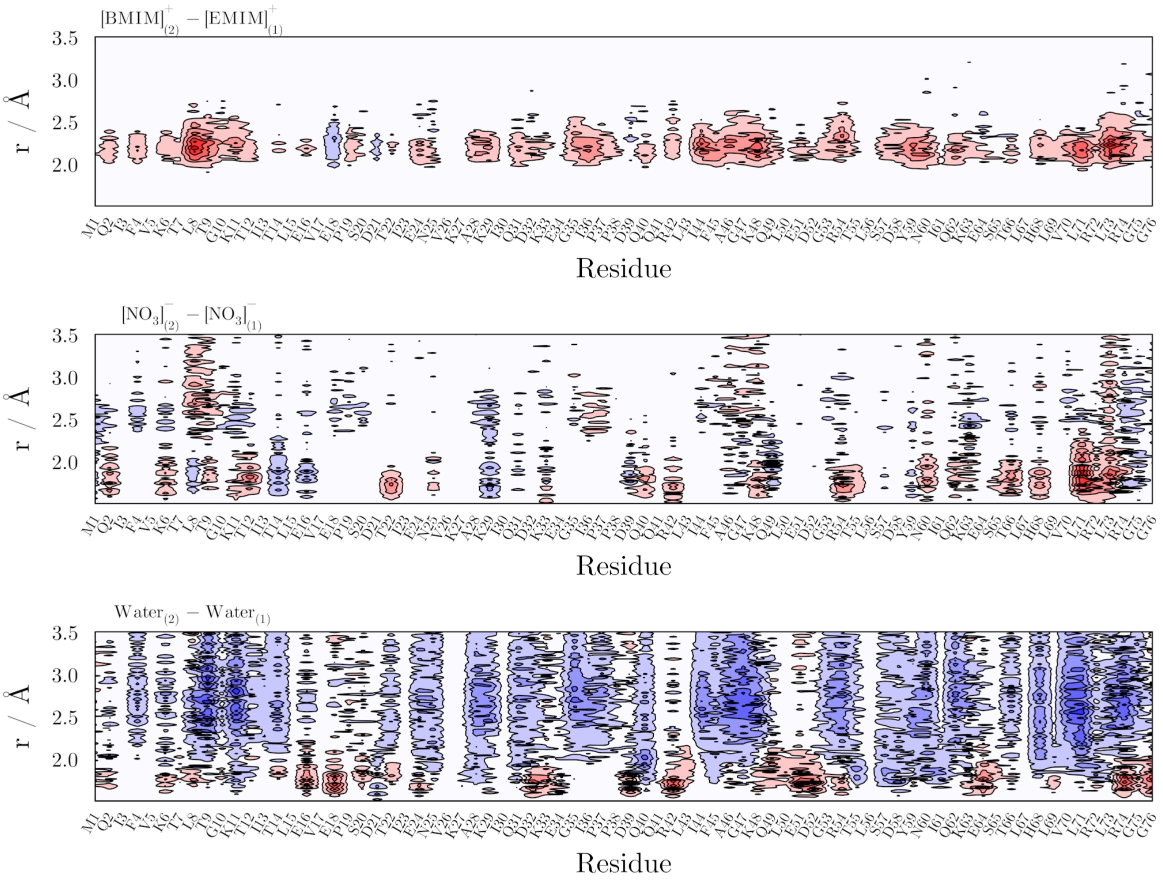

Figure S8 – Difference in protein residue contributions to the solvent-protein distribution functions for systems with $[BMIM]^+$ (denoted as underscore (2)) and $[EMIM]^+$ (denoted as underscore (1)). The density difference map compares the contributions of each protein residue to the MDDFs within 3.5 Å of the protein surface, based on data from 20 independent simulations for both cations. Red regions indicate a higher density of the solvent component in the $[BMIM]^+$ system, while blue regions indicate a higher density in the $[EMIM]^+$ system. The maps represent ~1.0 mol L^{-1} IL solutions with the anion $[NO]_4^-$.

Preferential hydration (Γ_{wp}) parameters

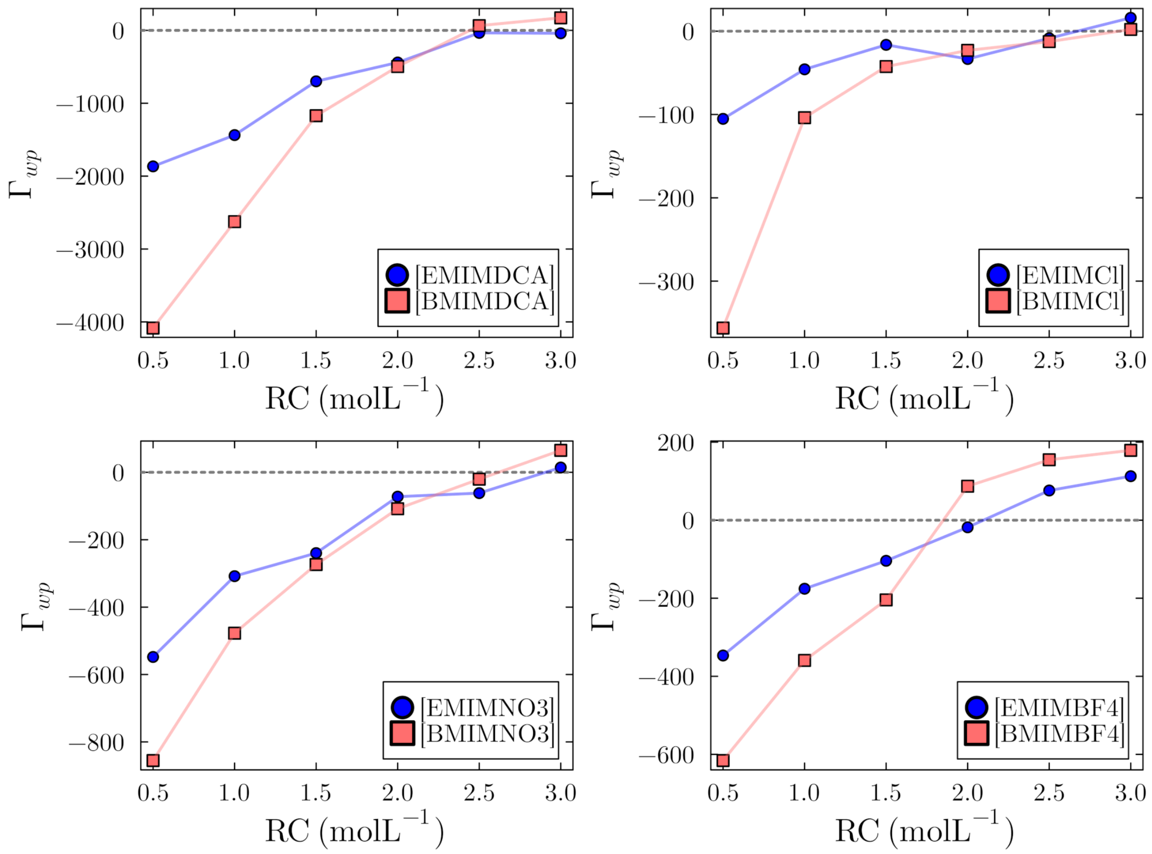

Figure S9 – Preferential hydration parameters of the protein (Γ_{wp}) for water relative to the ILs across different compositions and concentrations. The error bars represent the standard errors of the mean calculated from 20 simulation replicates. RC denotes the reference concentration, cited in the main text as C_{IL}.

Coordination number in the bulk solution.

Coordination numbers were computed using the **bulk_coordination()** function available in the MolSimToolkit.jl package. It computes the coordination number of one type of solvent molecule relative to another solvent molecule, as a function of the distance to a reference solute molecule.

Effectively, the maximum distance to the solute considered for a solvent molecule to be coordinated is set to 5 Å in the following plots.

The concentrations represent the actual bulk concentrations of the ionic liquids, calculated after system equilibration.

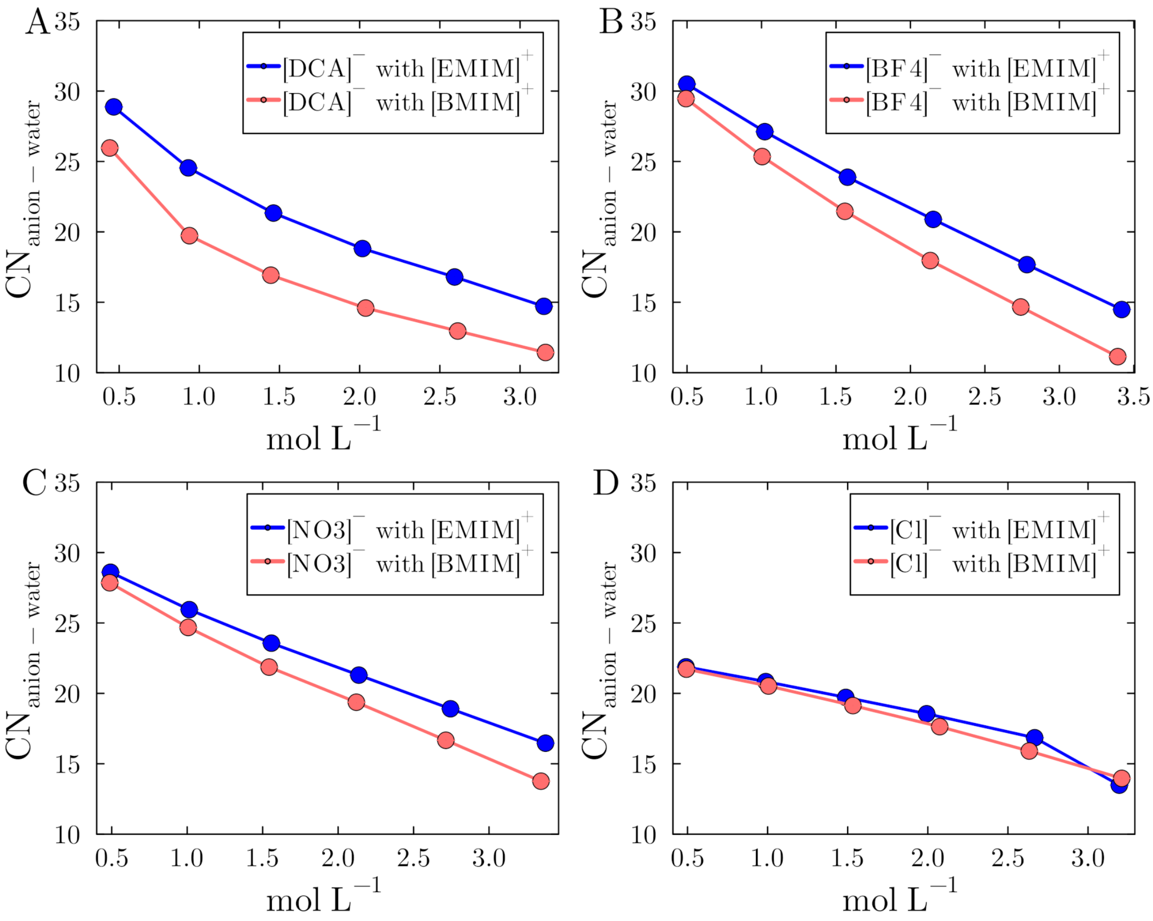

Figure S10 – Coordination number of water molecules around anions paired with $[EMIM]^+$ and $[BMIM]^+$. The coordination numbers represent the average number of water molecules within 5 Å of each anion in the bulk region, over all all effective IL concentrations.

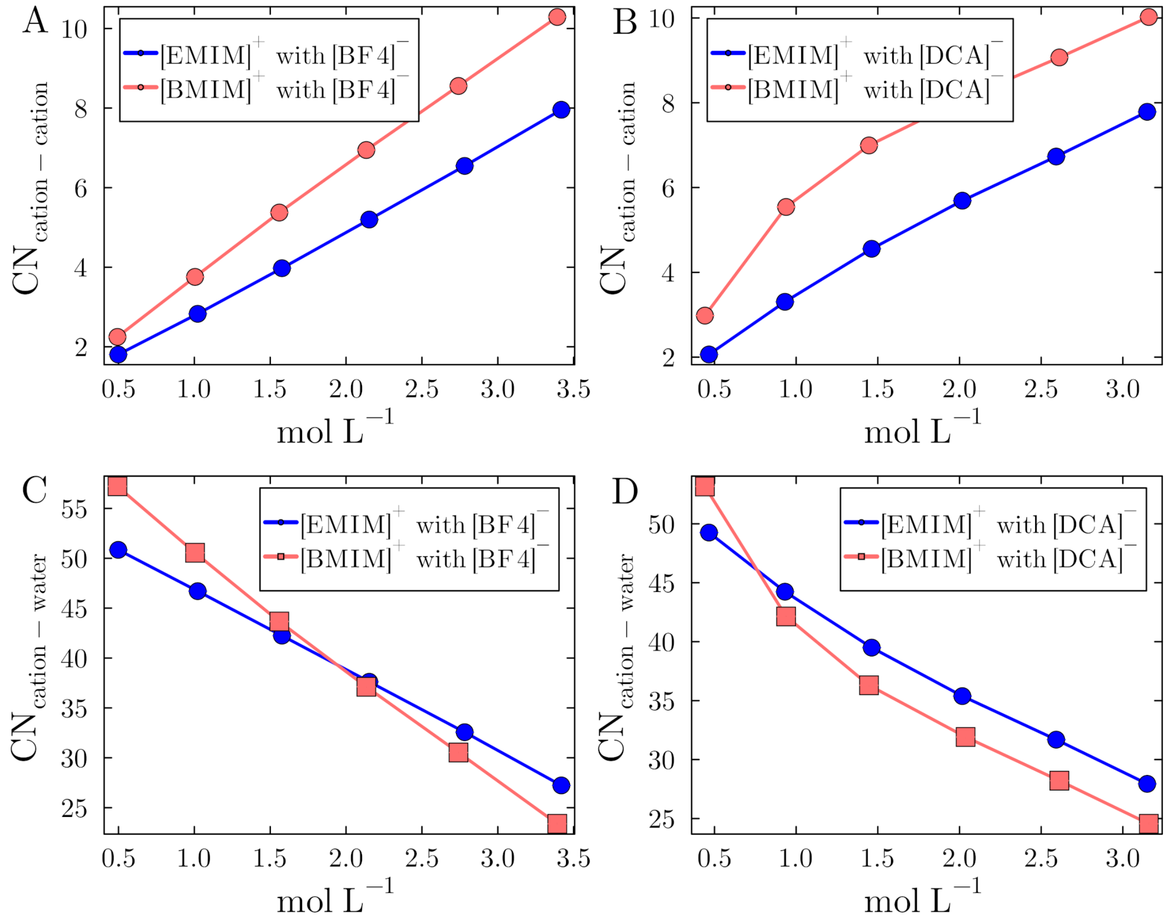

Figure S11 – Coordination numbers of cations and water molecules in the bulk region of ionic liquid systems with: **A)** Cation-cation coordination in systems containing the anion $[BF]_4^-$, **B)** Cation-cation coordination in systems containing the anion $[DCA]^-$, **C)** Water coordination around cations in systems paired with $[BF]_4^-$, and **D)** Water coordination around cations in systems paired with $[DCA]^-$. The coordination numbers are calculated within 5 Å of each species and evaluated in the bulk region across different ionic liquid concentrations.

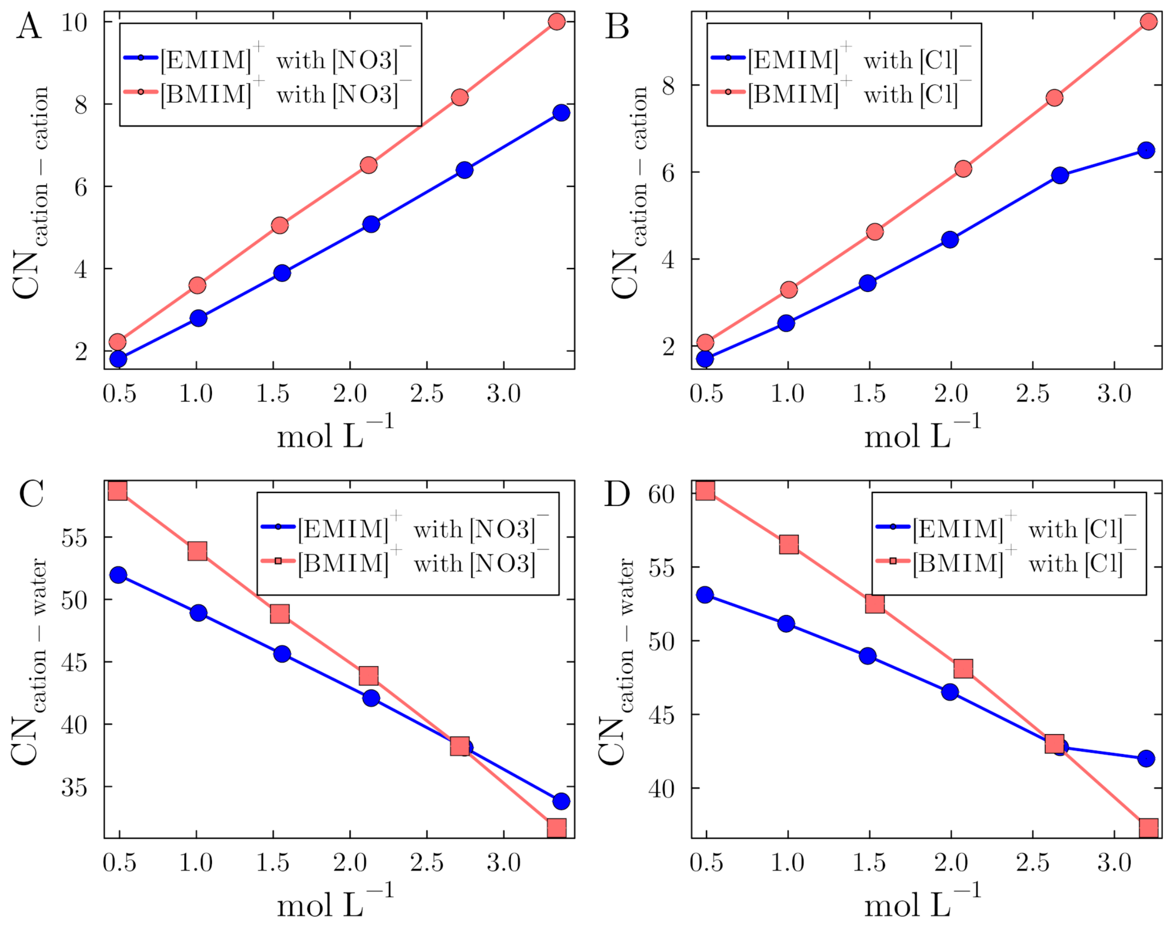

Figure S12 – Coordination numbers of cations and water molecules in the bulk region of ionic liquid (IL) systems with: **A)** Cation-cation coordination in systems containing the anion $[NO]_4^-$, **B)** Cation-cation coordination in systems containing the anion $[Cl]^-$, **C)** Water coordination around cations in systems paired with $[NO]_4^-$, and **D)** Water coordination around cations in systems paired with $[Cl]^-$. The coordination numbers are calculated within 5 Å of each species and evaluated in the bulk region across different IL concentrations.

Water distribution and Kirkwood-Buff integrals

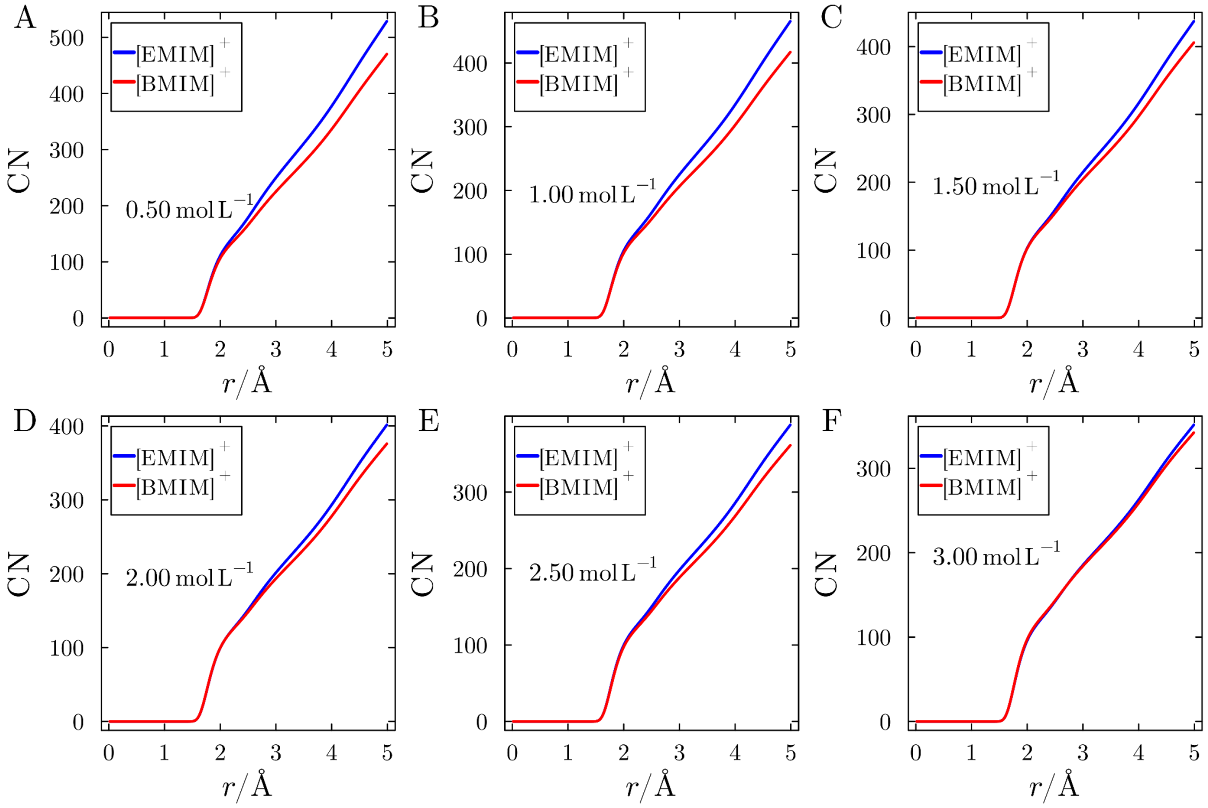

Figure S13: Coordination number of water up to 5 Å from the protein surface, at increasing concentration, for the systems with [EMIM][DCA] or [BMIM][DCA]. Panels A to F display the data across IL reference concentrations, C_{IL} ranging from 0.5 to 3.0 mol L^{-1}.

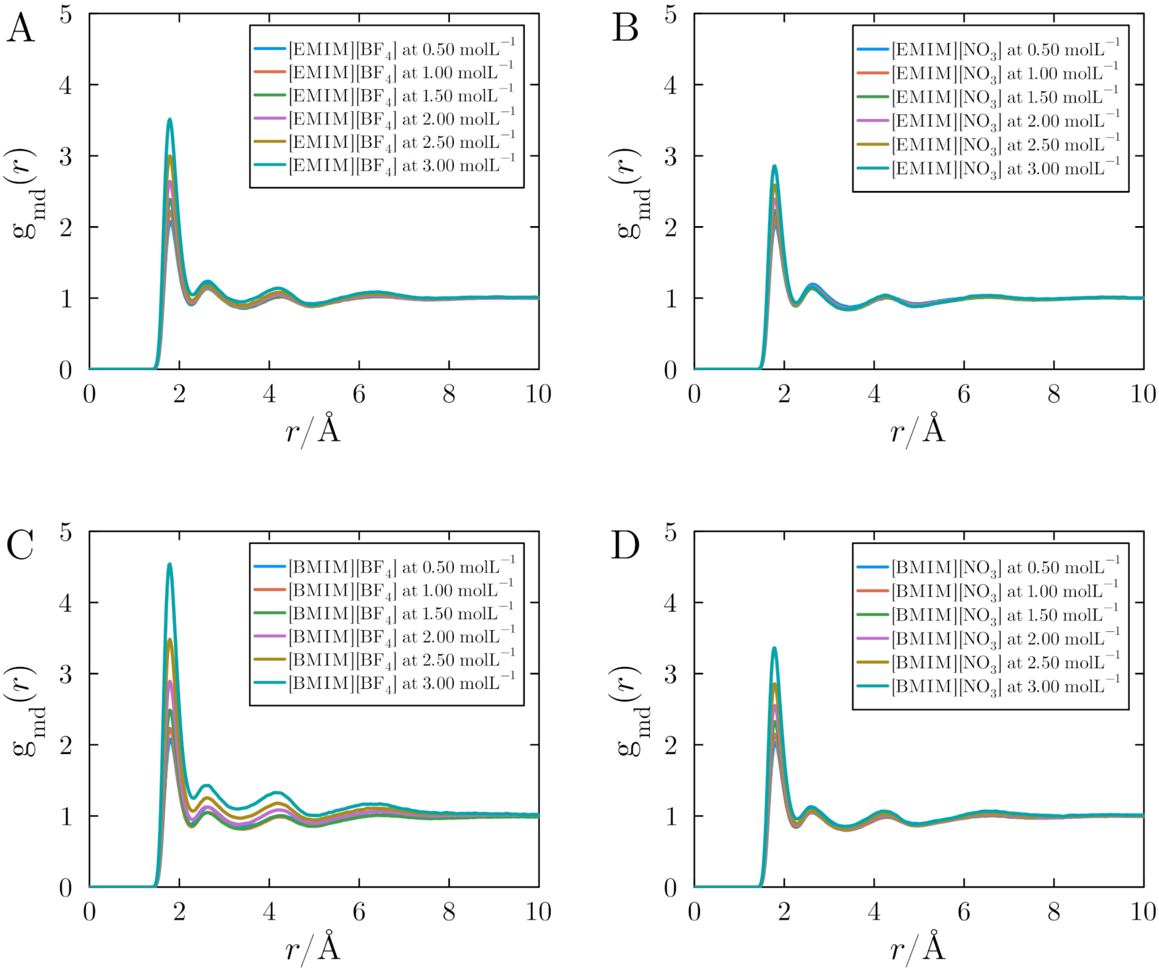

Figure S14 – Minimum-distance distribution functions (MDDFs) of water molecules around the protein in systems containing ionic liquids (ILs) with: **A)** [EMIM][BF]$_4$, **B)** [EMIM][NO]$_3$, **C)** [BMIM][BF]$_4$, and **D)** [BMIM][NO]$_3$. The figure highlights the spatial distribution of water molecules relative to the protein surface, evaluated across all simulated reference concentrations (in mol L^{-1}). Each curve represents the mean of 20 independent simulation runs, providing insights into water-protein interactions under varying ionic environments.

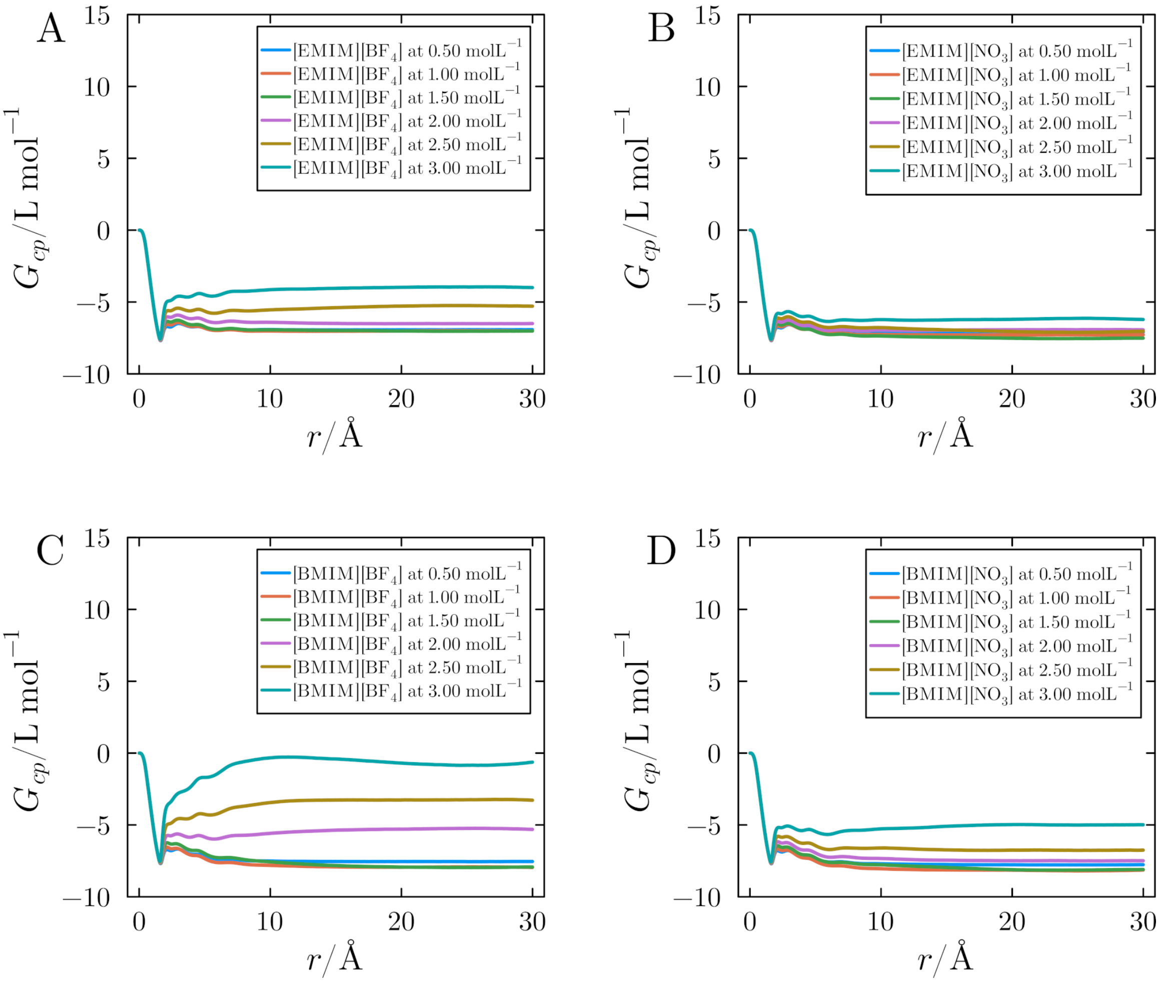

Figure S15 – Kirkwood-Buff integrals (KBIs) of water molecules in systems containing ionic liquids (ILs) with: **A)** [EMIM][BF]$_4$, **B)** [EMIM][NO]$_3$, **C)** [BMIM][BF]$_4$, and **D)** [BMIM][NO]$_3$. The figure presents the KBIs of water, evaluated across all simulated reference concentrations (in mol L^{-1}). Each curve represents the mean value calculated from 20 independent simulation runs, providing insights into the preferential solvation behavior and molecular organization of water in these IL systems.

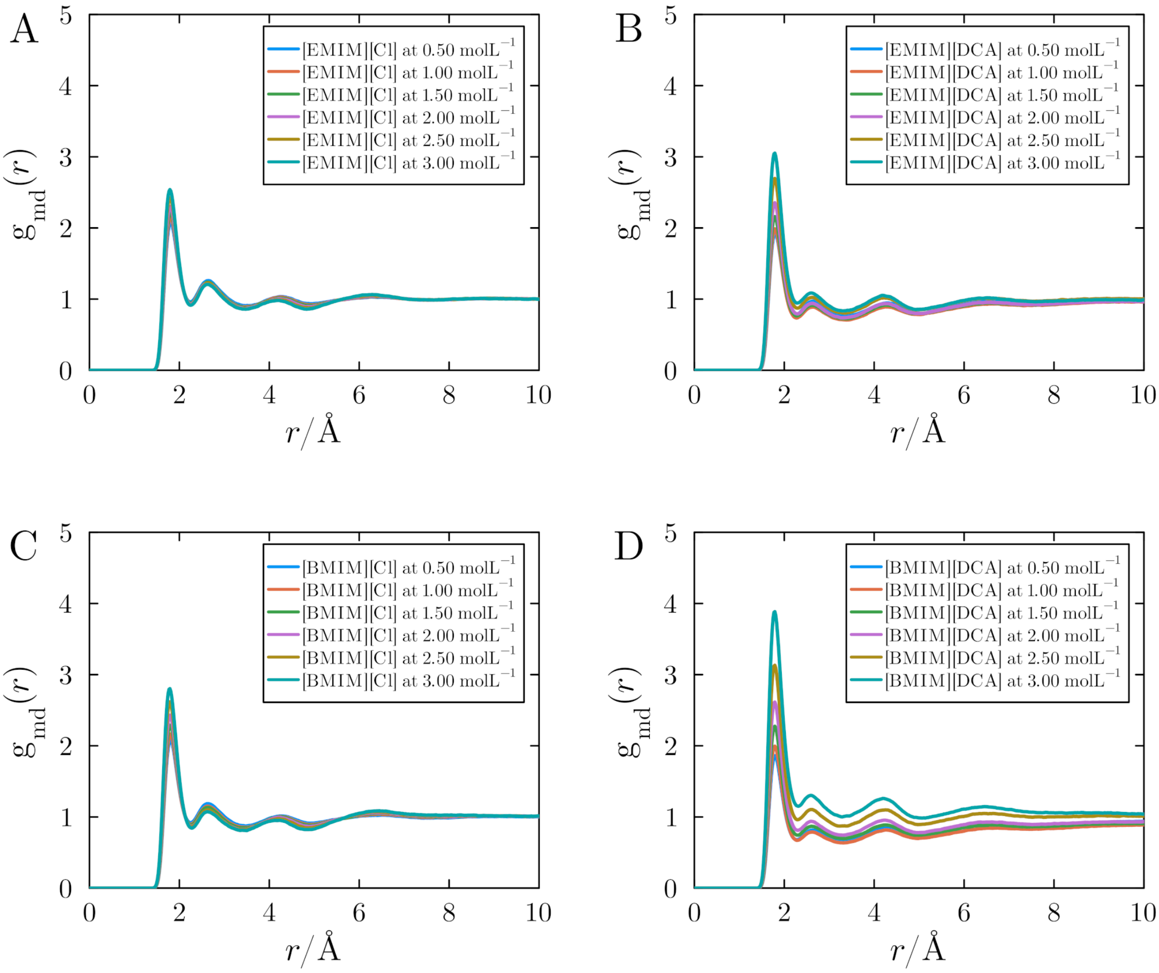

Figure S16 – Minimum-distance distribution functions (MDDFs) of water molecules around the protein in systems containing ionic liquids (ILs) with: **A)** [EMIM][Cl], **B)** [EMIM][DCA], **C)** [BMIM][Cl], and **D)** [BMIM][DCA]. The figure illustrates the spatial distribution of water molecules relative to the protein surface, evaluated across all simulated reference concentrations (in mol L^{-1}). Each curve represents the mean value calculated from 20 independent simulation runs, providing insights into water-protein interactions under varying ionic environments.

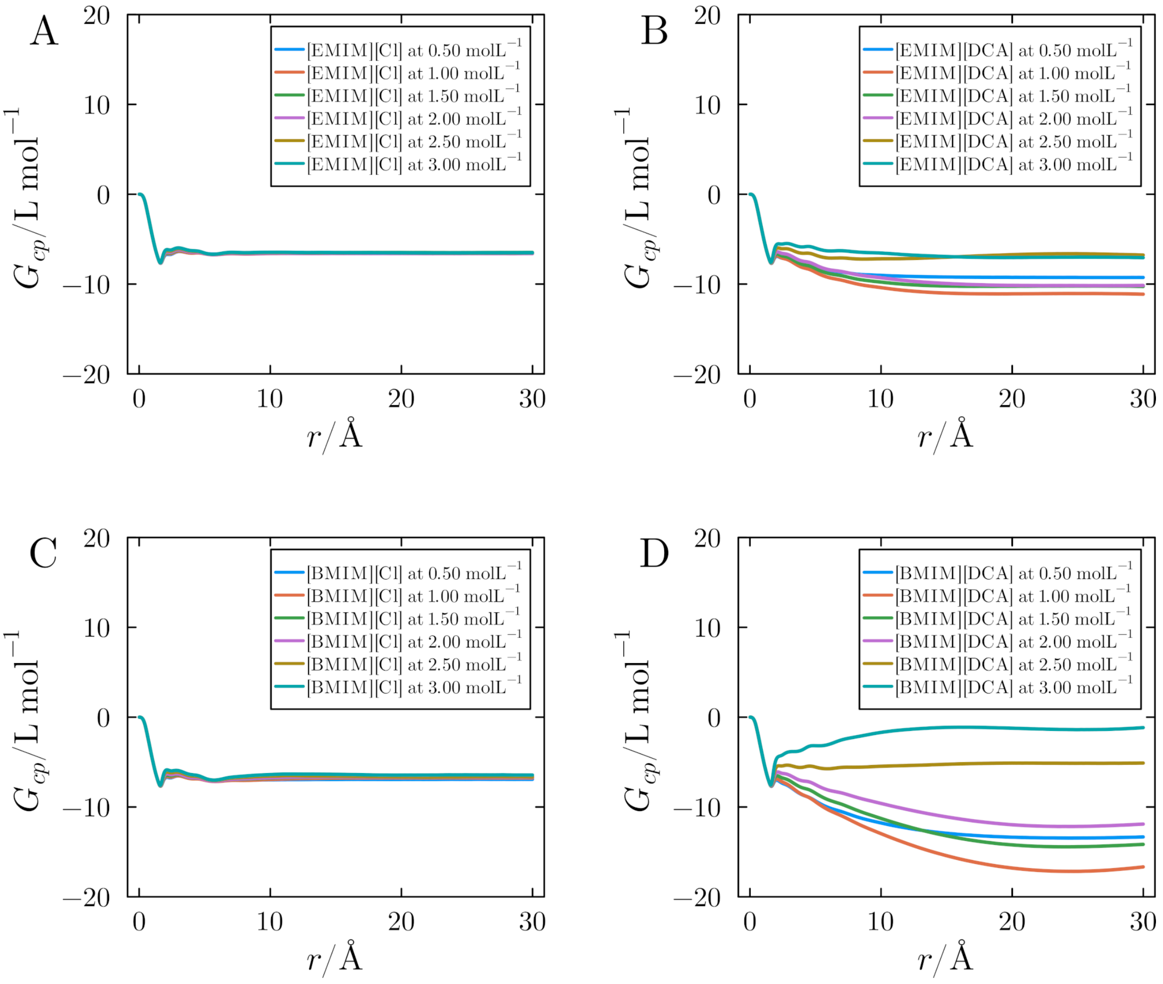

Figure S17 – Kirkwood-Buff integrals (KBIs) of water molecules in systems containing ionic liquids (ILs) with: **A)** [EMIM][Cl], **B)** [EMIM][DCA], **C)** [BMIM][Cl], and **D)** [BMIM][DCA]. The figure presents the KBIs of water, evaluated across all simulated reference concentrations (in mol L^{-1}). Each curve represents the mean value calculated from 20 independent simulation runs, offering insights into the preferential solvation and molecular organization of water in these IL systems.

Protein structural variability

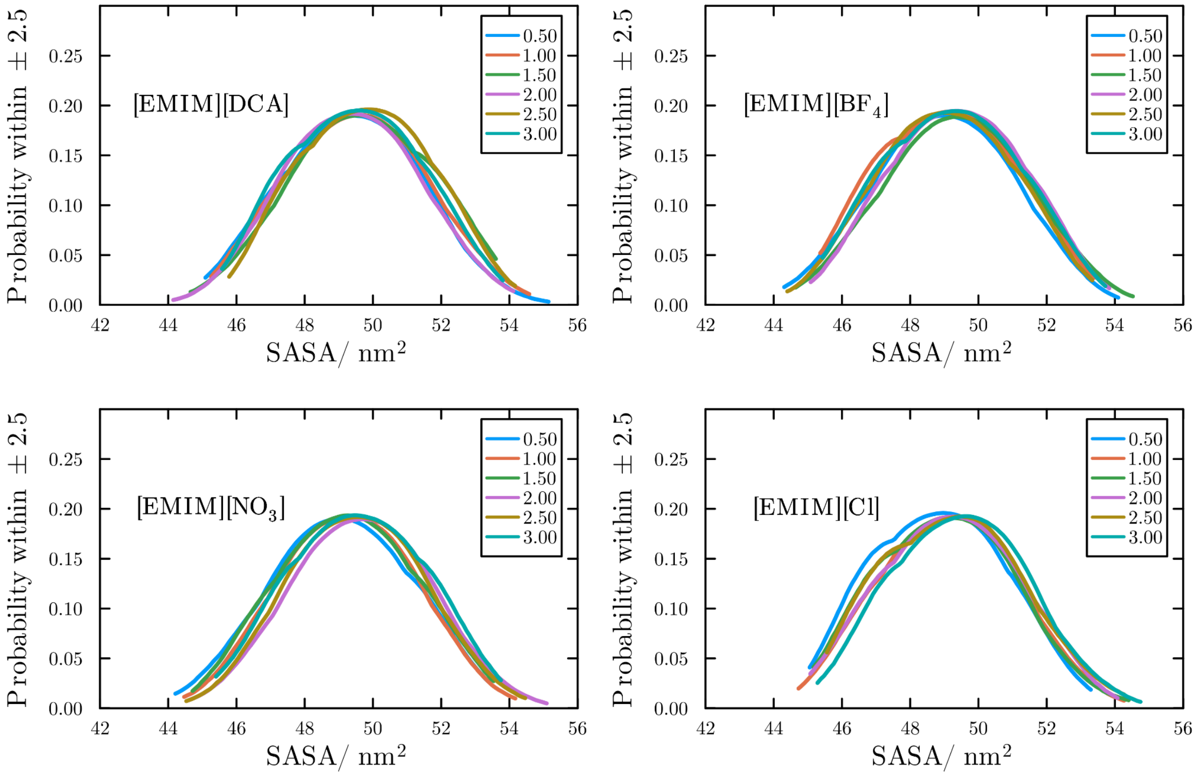

Figure S18 – Distribution of the solvent-accessible surface area (SASA) for ubiquitin in EMIM-based systems. Similar distributions indicate similar exposure of the protein surface to the solvent and structural stability in all simulations.

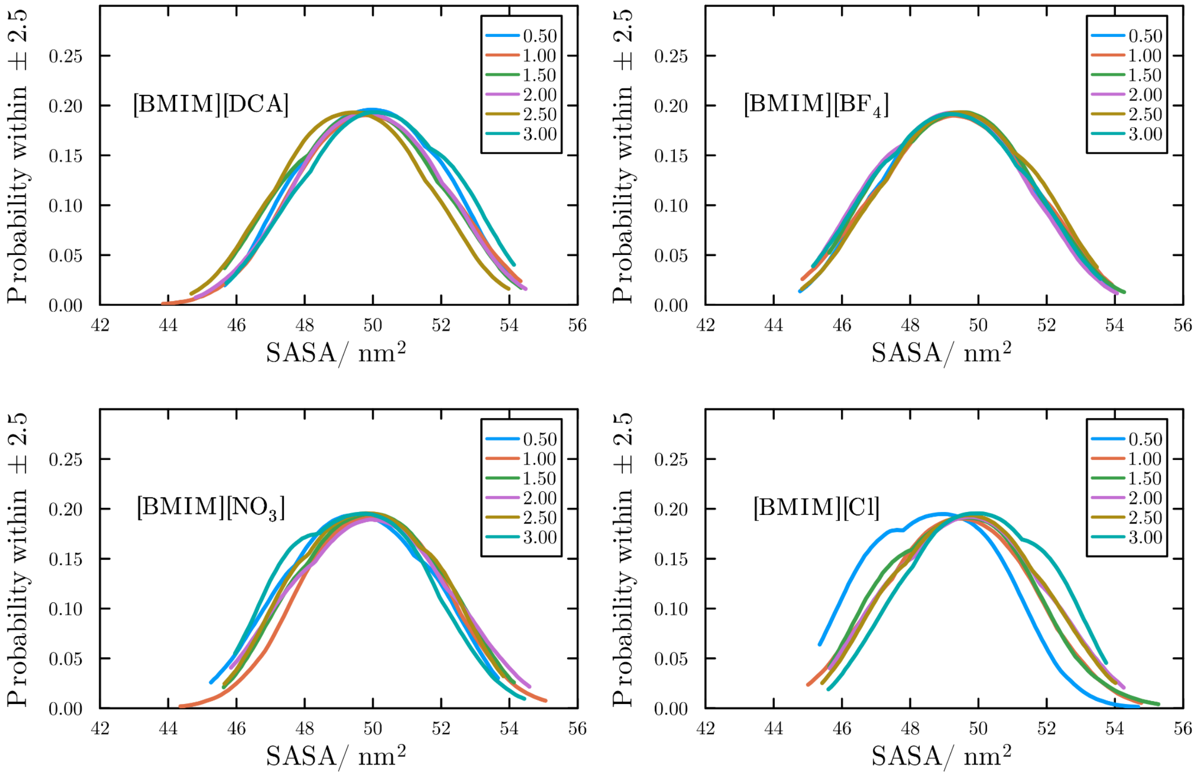

Figure S19 – Distribution of the solvent-accessible surface area (SASA) for ubiquitin in BMIM-based systems. Similar distributions indicate similar exposure of the protein surface to the solvent and structural stability in all simulations.

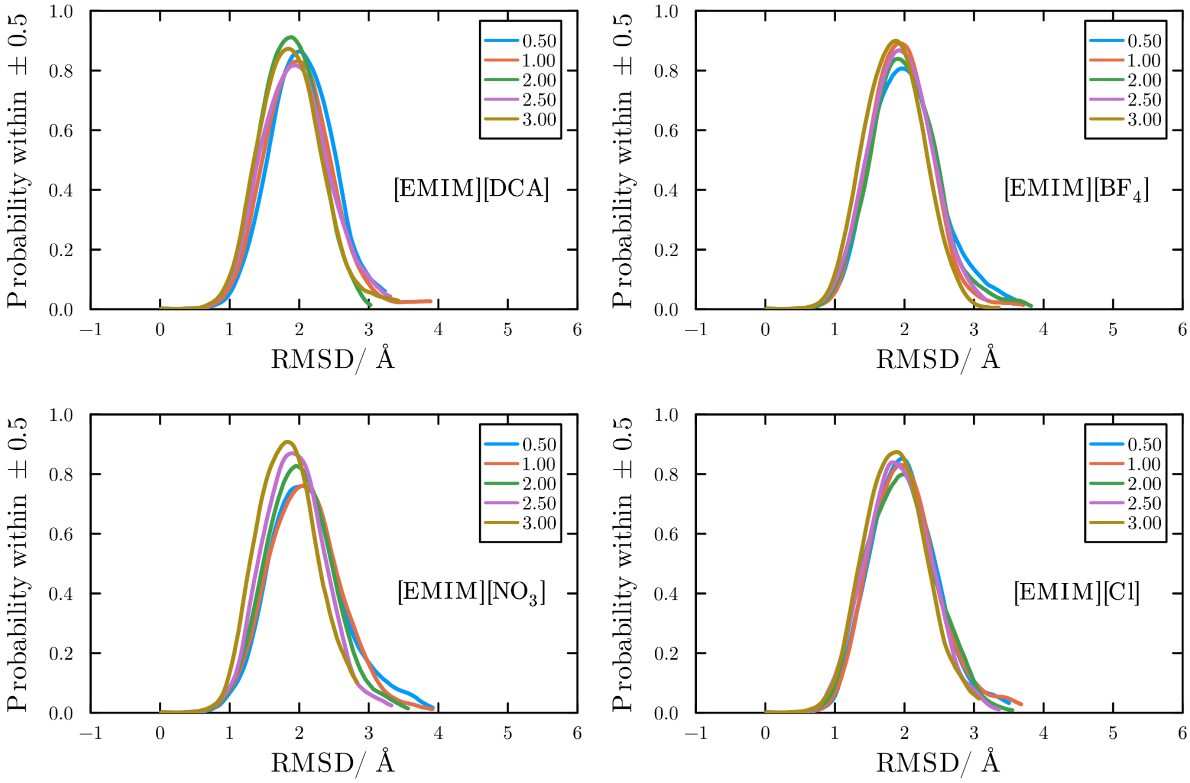

Figure S20 – Backbone RMSD distribution for ubiquitin in EMIM-based systems. The distributions are similar and centered around 2Å, supporting the conclusion of structural preservation during the simulations.

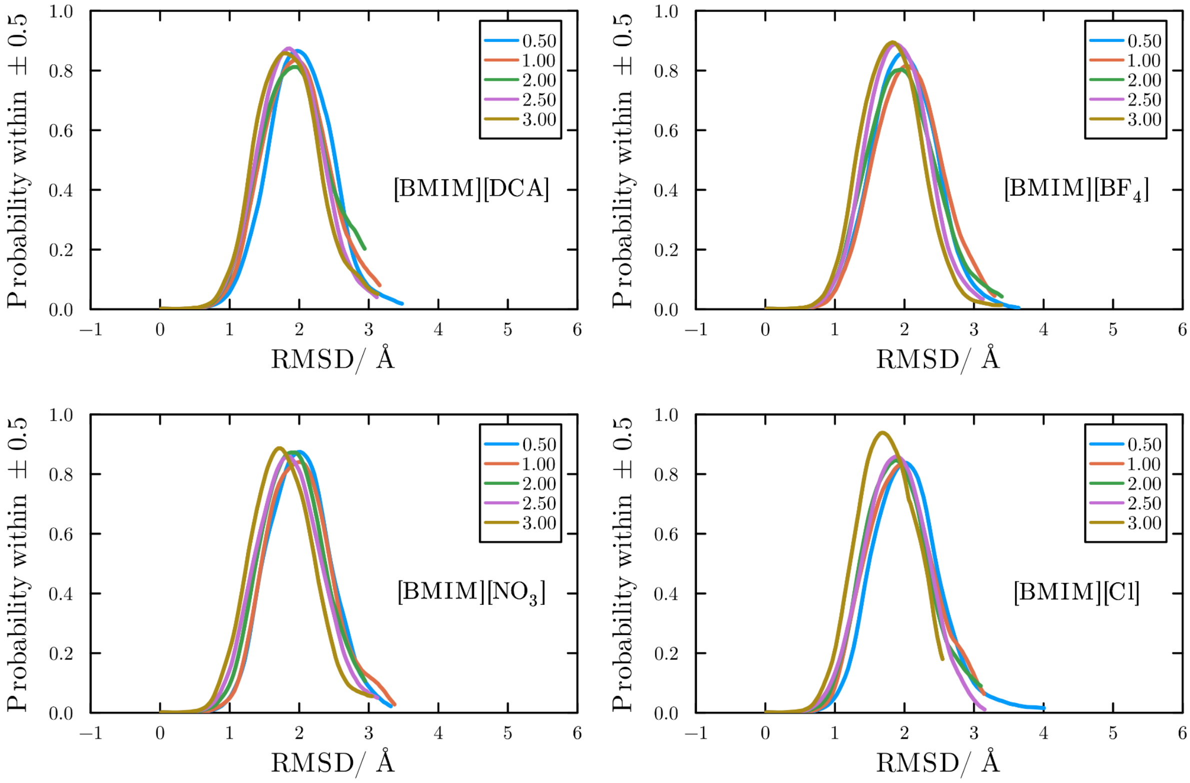

Figure S21 – Backbone RMSD distribution for ubiquitin in BMIM-based systems. The distributions are similar and centered around 2Å, supporting the conclusion of structural preservation during the simulations.
